# *SPP1*+ macrophages promote head and neck squamous cell carcinoma progression by secreting TNF-α and IL-1β

**DOI:** 10.1186/s13046-024-03255-w

**Published:** 2024-12-26

**Authors:** Chun Liu, Kun Wu, Chuwen Li, Zhen Zhang, Peisong Zhai, Haiyan Guo, Jianjun Zhang

**Affiliations:** 1 Department of Oral and Maxillofacial-Head and Neck Oncology, Shanghai Ninth People’s Hospital, Shanghai Jiao Tong University School of Medicine, Shanghai, China; 2 College of Stomatology, Shanghai Jiao Tong University, Shanghai, China; 3 National Center for Stomatology, Shanghai Jiao Tong University, Shanghai, China; 4 National Clinical Research Center for Oral Diseases, Shanghai Jiao Tong University, Shanghai, China; 5 Shanghai Key Laboratory of Stomatology, Shanghai, China; 6 Shanghai Research Institute of Stomatology, Shanghai Jiao Tong University, Shanghai, China; 7 Shanghai Center of Head and Neck Oncology Clinical and Translational Science, Shanghai Jiao Tong University, Shanghai, China; 8 Department of Oral and Maxillofacial Surgery, Second Xiangya Hospital of Central South University, Changsha, China; 9 Department of Clinical Laboratory, Shanghai Ninth People’s Hospital, Shanghai Jiao Tong University School of Medicine, Shanghai, China

**Keywords:** Macrophages, Single-cell RNA sequencing, SPP1 + macrophages, TNF-α; IL-1β

## Abstract

**Background:**

Head and neck squamous cell carcinoma (HNSCC) is a very aggressive disease characterized by a heterogeneous tumor immune microenvironment (TIME). Tumor-associated macrophages (TAMs) constitute the major innate immune population in the TIME where they facilitate crucial regulatory processes that participate in malignant tumor progression. SPP1 + macrophages (SPP1 + Macs) are found in many cancers, but their effects on HNSCC remain unknown. This study aimed to identify and validate the role and function of SPP1 + Macs in the malignant progression of HNSCC.

**Methods:**

In this study, we applied single-cell RNA sequencing (scRNA-seq) analyses of paired tumor and normal tissues from 5 HNSCC patients to identify tumor-specific SPP1 + Macs. RT-qPCR and multiplex immunohistochemical and multiplex immunofluorescence staining were used to verify the presence of SPP1 + Macs in the clinical samples. Gene set variation analysis suggested that SPP1 + Macs were actively involved in cytokine production. Thus, we constructed SPP1-OE macrophages and SPP1-KD macrophages (both differentiated from THP-1 cells), performed a Luminex liquid suspension chip detection assay to detect differential cytokines, and further assessed their biological functions and mechanisms in several HNSCC cell lines and adjacent macrophages. An in vivo experiment was used to verify the function of SPP1 + Macs in HNSCC progression.

**Results:**

The scRNA-seq results revealed that myeloid cells were heterogeneous and strongly correlated with tumor cells in the TIME in HNSCC and identified tumor-specific SPP1 + Macs, which were positively correlated with poor prognosis of HNSCC patients. Gene set variation analysis (GSVA) suggested that SPP1 + Macs were actively involved in cytokine production. Luminex liquid suspension chip detection assay indicated that SPP1 + Mac-derived TNF-α and IL-1β played important roles. Both in vitro and in vivo experiments and the use of VGX-1027, an inhibitor of macrophage-derived TNF-α and IL-1β, confirmed that SPP1 + Mac-derived TNF-α and IL-1β promoted HNSCC progression by supporting tumor cell proliferation and migration. Mechanistically, we found that TNF-α and IL-1β were upregulated due to NF-kappa B signaling pathway activation in SPP1 + Macs. Moreover, SPP1 + Mac-derived TNF-α and IL-1β promoted the expression of OPN in both tumor cells and other adjacent macrophages through different signaling pathways.

**Conclusions:**

SPP1 + Macs increase the secretion of TNF-α and IL-1β via the NF-kappa B pathway to promote HNSCC cell proliferation, and TNF-α and IL-1β in turn upregulate the expression of OPN in tumor cells and macrophages; thus, SPP1 + Macs may be a candidate target through which antitumor efficacy can be enhanced.

**Supplementary Information:**

The online version contains supplementary material available at 10.1186/s13046-024-03255-w.

## Background

Head and neck squamous cell carcinoma (HNSCC), of which approximately 900,000 new cases occur every year, is responsible for around 500,000 deaths annually and is one of the most common cancers worldwide [1, 2]. Despite advancements in various treatment modalities (including surgical resection, adjuvant radiation, chemotherapy and immunotherapy), the overall survival rate of HNSCC patients is approximately 50% [3]. Thus, understanding the molecular mechanisms of HNSCC initiation and progression is crucial for developing more appropriate treatment strategies.

Macrophages are key components of the tumor immune microenvironment (TIME), where they perform multiple functions, are strongly associated with prognosis and exhibit subset heterogeneity in different cancer types [4, 5]. Macrophages are traditionally classified as M1-like or M2-like macrophages. However, recent studies revealed that in almost all cancers, both M1 and M2 gene signatures could be identified in the same macrophage subpopulation. What’s more, a recent study focused on HNSCC suggested that CXCL9-SPP1 might be a better classification system to define the polarity and prognosis than the conventional M1 and M2 subtypes [6]. Using single-cell RNA sequencing (scRNA-seq) and spatial transcriptomics techniques, more detailed and specific macrophage subtypes have been identified in efforts to modernize macrophage-targeted therapies. Increasing numbers of studies suggest that tumor-associated macrophages (TAMs) contribute to immunosuppressive properties in cancers; for example, interferon-primed TAMs, immune regulatory TAMs and inflammatory cytokine-enriched TAMs (Inflam-TAMs) have been shown to recruit and regulate immune cells to suppress the immune response and drive tumor progression [7, 8].

Secreted phosphoprotein 1 (SPP1), encoded by the SPP1 gene, is also known as osteopontin (OPN) and has been shown to alleviate fibrosis and to be positively correlated with malignancy and chemoresistance in multiple cancers [9, 10]. Recent studies have indicated that SPP1 + macrophages (SPP1 + Macs) constitute a common TAM cluster in various cancer types and that SPP1 is a marker of Inflam-TAMs and proangiogenic TAMs (Angio-TAMs). In colorectal cancer (CRC) and gastric carcinoma, Inflam-TAMs, which promote inflammatory cytokine production, may recruit immune cells, including monocytes and lymphocytes, during inflammation [7, 11]. Angio-TAMs promote tumor cell epithelial–mesenchymal transition (EMT) and angiogenesis and are associated with poor prognosis via a mechanism mediated by NF-kappa B signaling, Notch signaling or VEGF signaling pathways in many tumor types, including breast cancer, CRC, gastric carcinoma, hepatocellular carcinoma (HCC), non-small cell lung cancer and pancreatic ductal adenocarcinoma (PDAC) [7, 12–14]. However, the mechanisms and functions of SPP1 + Macs in HNSCC remain unclear.

Here, we obtained scRNA-seq data from 5 pairs of HNSCC clinical samples and revealed that tumor-specific SPP1 + Macs were correlated with poor prognosis. We found that SPP1 + Macs promoted HNSCC cell proliferation and migration through the secretion of the cytokines tumor necrosis factor alpha (TNF-α) and interleukin-1 beta (IL-1β). In addition, SPP1 + Mac-derived TNF-α and IL-1β promoted the expression of OPN in both tumor cells and macrophages. Both SPP1 knockdown in macrophages and treatments aimed at reducing the release of macrophage-derived cytokines inhibited HNSCC cell proliferation and migration. The aim of this study was to investigate the role and mechanism of SPP1 + Macs in HNSCC cells to identify potential therapeutic targets for HNSCC.

## Methods and materials

### Ethics statement

Written informed consent was obtained from all participants before starting the experiments. All experimental methods abided by the Helsinki Declaration. This study was approved by the Ethics Committee of the Ninth People’s Hospital, Shanghai Jiao Tong University School of Medicine (Shanghai, China). All animal experiments were performed in accordance with the National Institutes of Health (NIH) Guide for Care and Use of Laboratory Animals, with the approval of Ninth People’s Hospital, Shanghai Jiao Tong University School of Medicine Institutional Animal Care and Use Committee.

### Patients and specimens

All clinical HNSCC samples and paired adjacent normal tissue samples were obtained from the Department of Oral and Maxillofacial-Head & Neck Oncology, Shanghai Ninth People’s Hospital, Shanghai Jiao Tong University School of Medicine. Seventy-four tumor tissues and paired normal tissues for real-time reverse transcription PCR analysis, IHC staining and mIHC staining were collected from HNSCC patients and healthy donors between March 2017 and December 2023. The World Health Organization Classification of Tumors and the American Joint Committee on Cancer (AJCC) tumor–node–metastasis (TNM) staging system (8th edition) were used to determine the pathological differentiation status and clinical stage. The detailed characteristics of the 74 pairs HNSCC patients and healthy participants are listed in Table S1.

### Dissociation of HNSCC samples

The five HNSCC tumor samples and paired adjacent normal samples were washed in ice-cold storage buffer (RPMI-1640 medium + 0.04% BSA) and cut into pieces with a volume of approximately 0.5 mm^3^. The tissue pieces were then digested with a human Tumor Dissociation Kit (Miltenyi) according to the manufacturer’s instructions, and the lysates were filtered through 40 μm cell strainers. Before the addition of 1X red blood cell lysis buffer (MACS buffer), the cells were centrifuged at 300 × g for 5 min at 4 °C, and the supernatants were discarded. After incubation in MACS buffer at 4 °C for 10 min, the cells were pelleted by two rounds of centrifugation at 300 × g for 5 min at 4 °C. Finally, the cells were washed and resuspended in RPMI-1640 medium. The detailed information of the 5 HNSCC patients are listed in Table S2.

### Single-cell RNA sequencing (scRNA-seq) and data processing

Following the 10 × Genomics protocol, freshly prepared cell suspensions were used to construct cDNA libraries with 10 × Genomics Chromium Next GEM Single Cell 3ʹ Reagent Kits v3.1, and sequencing was performed on the Illumina NovaSeq 6000 platform in PE150 mode.

The Cell Ranger software pipeline (version 3.1.0) was used for barcode demultiplexing. Loupe Browser 7 was used to identify cell types and cell subtypes. The cell communication analysis was performed using the CellChat (v 1.1.3) R package.

### TCGA database analysis

Gene expression and clinical data were downloaded from TCGA HNSCC database and filtered into two subgroups with normal (122 cases) and primary tumor (377 cases) and normal (44 cases) and primary tumor (520 cases) [14, 15]. Differences in SPP1 expression between normal and primary tumor in HNSCC were compared using t-test.

To elvalute the correlation between SPP1 + Macs and related patient survival rate, Cibersortx deconvolution was used to deduce the ratio of SPP1 + Mac of each TCGA sample. Basically the expression matrix of our annotated single cell data was used to as a cell type reference profile generate a signature matrix, the the cibersortx was adopted to calculate the proportion of each cell type from the bulk sequencing data of TCGA. Then the TCGA samples were divided into SPP1 + TAM high and low group to do the survival analysis.

### Cell lines and culture

The HNSCC cell lines HN6 and CAL27 were used in this study. HN6 cells were kindly provided by the University of Maryland Dental School, USA. CAL27 cells were purchased from the American Type Culture Collection (ATCC; USA). All cells were cultured in Dulbecco’s modified Eagle medium (Basal Media) supplemented with 10% fetal bovine serum (FBS; Gibco), penicillin (100 units/mL, New Cell & Molecular Biotech), and streptomycin (100 mg/mL, New Cell & Molecular Biotech) in a humidified 5% CO2 atmosphere at 37 °C. All cells were routinely tested for mycoplasma contamination with the GMyc-PCR Mycoplasma Test Kit (Yeasen).

### Macrophage culture and treatment

Macrophages were differentiated from THP-1 cells, a human monocytic leukemia cell line kindly provided by Guangzhou ELGBIO Company. THP-1 cells were cultured in RPMI-1640 medium (Basal Media) supplemented with 10% FBS (Gibco) at 37 °C in a humidified 5% CO2 atmosphere. To differentiate THP-1 cells into macrophages, THP-1 cells were cultured with phorbol 12-myristate 13-acetate (PMA; 100 ng/mL; MCE) in RPMI-1640 medium without FBS for 48 h.

### Lentiviral transduction and screening for stable cell lines

The SPP1 overexpression, SPP1 knockdown, and control lentiviral vectors were constructed by Genomeditech Biotechnology Co., Ltd. The SPP1-knockdown (SPP1-KD), SPP1-overexpressing (SPP1-OE), and control (SPP1-NC and SPP1-Sh-NC) were generated by lentiviral transduction according to the manufacturer’s protocol. The cells with stable expression were identified by screening with culture medium supplemented with puromycin (Yeasen) at a final concentration of 10 μg/mL after 2 weeks. The sequences used for the overexpression and knockdown of specific targets are listed in Table S3.

### RNA extraction and real-time PCR analysis

For tissue RNA extraction, total RNA from clinical patient samples was extracted using TRIzol reagent (Takara) and reverse transcribed into cDNA using a PrimeScript RT Reagent Kit (Takara).

For cellular RNA extraction, transfected macrophages (differentiated from THP-1 cells) were seeded in 6-well plates for 48 h. RNA was extracted with the RNA-Quick Purification Kit (ESunbio) according to the manufacturer’s protocol. Reverse transcription was conducted with Evo M-MLV RT Master Mix (Accurate Biology). Real-time PCR was conducted with Hieff qPCR SYBR Green Master Mix (Yeasen) in a StepOnePlus Real-time PCR System. The 2^−ΔΔCt^ method was used to normalize the expression of target genes to that of β-actin. The sequences of the primers used in this study are listed in Table S3.

### Western blot analysis

Transfected THP-1 cells were seeded in 10-cm plates (5 × 10^6^ cells per plate) and treated with PMA. After 48 h of treatment, the culture medium was discarded, and the cells were washed with ice-cold PBS 2 times. Then, SEMS lysis buffer (Beyotime) was added to the plates at the indicated times. After heating at 105 °C for 15 min, the protein concentrations in the cell lysates were measured with a BCA Assay Kit (New Cell & Molecular Biotech). Protein samples (20 μg) were loaded on SurePAGE protein gels (GenScript) before separation of proteins at 150 V for 70 min. Then, the proteins in the gel were transferred to a 0.22-μm polyvinylidene fluoride membrane (Millipore). The membrane was then blocked with 5% skim milk in TBST (Solarbio) at room temperature and incubated with primary antibodies at 4 °C overnight. After being washed six times with TBST, the membrane was incubated with the corresponding secondary antibodies for 1 h at room temperature. Finally, enhanced chemiluminescence reagents (New Cell & Molecular Biotech) were used to visualize the signals with an Amersham Imager 600. The antibodies used are listed in Table S4. The densitometry analysis of the blots was calculated by ImageJ.

### Immunohistochemical (IHC) staining

Paraffin sections of HNSCC clinical samples, adjacent normal tissues, and tumors from mice were heated at 65 °C for 2 h before being dewaxed by three rounds of immersion in 100% v/v dimethylbenzene; sequential immersion in 100%, 90%, and 70% v/v ethanol; and immersion in pure water. Tris–EDTA antigen retrieval buffer (Proteintech) was used for heat-induced epitope retrieval in a microwave oven. Then, 3% v/v H2O2 (Absin) was applied for 15 min to block endogenous peroxidase activity, and 10% v/v goat serum (Biosharp) was used for Fc blocking. Primary antibodies were diluted and applied to the tissue sections for incubation at 4 °C overnight. A goat anti-mouse/rabbit poly-horseradish peroxidase (HRP)-conjugated secondary antibody (Proteintech) was applied to the tissue sections after they were washed 5 times with PBS. A DAB detection kit (Absin) was used for visualization. Nuclei were counterstained with hematoxylin (Sangon Biotech). After dehydration by sequential immersion in 70%, 90%, and 100% v/v ethanol and three rounds of immersion in 100% v/v dimethylbenzene, the cover slips were mounted with neutral balsam (Absin), and the slides were scanned with a Vectra automated quantitative pathology system. The antibodies used are listed in Table S4.

### Multiplex immunofluorescence

Dewaxing and antigen epitope retrieval were performed on paraffin sections of HNSCC samples and mouse tumors as described above for IHC staining. Next, 10% v/v goat serum was used for Fc blocking. The sections were then incubated with primary antibodies at 4 °C overnight. Before being incubated with fluorophore-conjugated secondary antibodies (Cell Signaling Technology) at room temperature for 1 h, the sections were washed with PBST 5 times. After another PBST wash, the membrane was incubated with another primary antibody. Nuclei were stained with 4’,6-diamidino-2-phenylindole (DAPI; Beyotime) for 15 min. All coverslips were mounted with Fluoromount-G (Beyotime). Fluorescence micrographs were acquired with a Zeiss LSM900 microscope, and the images were further processed with ZEN 1.1.0. The antibodies used are listed in Table S4.

### Multiplex immunohistochemical (mIHC) staining

Dewaxing, antigen epitope retrieval and Fc blocking were performed on paraffin sections of HNSCC samples and mouse tumors as described above for IHC staining. Next, the sections were then incubated with primary antibodies at 4 °C overnight. After being incubated with goat anti-mouse/rabbit HRP-conjugated secondary antibody (Servicebio) at room temperature for 20 min, fluorescent probes target HRP (MCE) was used to incubate sections at room temperature for another 20 min, the sections were washed with PBST 5 times and heated for antigen epitope retrieval for next round of primary antibody staining. After staining all markers, nuclei were stained with DAPI for 15 min. All coverslips were mounted with Fluoromount-G (Beyotime). Fluorescence micrographs were acquired with a Zeiss LSM900 microscope, and the images were further processed with ZEN 1.1.0. The antibodies used are listed in Table S4.

### Immunocytochemistry (ICC)

Transfected macrophages (differentiated from THP-1 cells) were seeded (5 × 10^3^ cells/well) in confocal dishes. After 48 h of induction, the macrophages were treated with PDTC or serum-free medium for 1 h. The cells were then washed with precooled PBS 2 times and fixed with 4% paraformaldehyde (PFA; Biosharp) for 15 min. Triton X-100 (0.1% v/v; Solarbio) was used to permeabilize the cells, and 5% v/v goat serum in TBST was used for Fc blocking. The cells were incubated with primary antibodies at 4 °C overnight, washed with PBS 2 times and incubated with secondary antibodies for 1 h in the dark at room temperature. Nuclei were stained with DAPI for 15 min. Images of the cells were acquired with a Zeiss LSM900 microscope and further processed with ZEN 1.1.0. The antibodies used are listed in Table S4.

### Luminex liquid suspension chip detection assay

Detection of inflammatory cytokines and chemokines via a Luminex liquid suspension chip detection assay was performed by Wayen Biotechnologies. The Bio-Plex Pro Human Chemokine Panel 40-plex Kit was used in accordance with the manufacturer's instructions with the Luminex 200 system. In brief, macrophages and SPP1 + Macs (both differentiated from THP-1 cells) were treated with serum-free RPMI-1640 medium for 24 h after being treated with PMA for 48 h. The supernatants were collected for further analysis. Samples (50 µL) from the standard, experimental and blank groups were added to microbead-coated 96-well plates, incubated for one hour and then incubated with the detection antibody for 30 min. Subsequently, streptavidin-PE was added to each well for 10 min, and the values were determined using the Bio-Plex MAGPIX System.

### Enzyme-linked immunosorbent assay (ELISA)

Transfected macrophages (differentiated from THP-1 cells) were seeded in 6-well plates (1 × 10^6^ cells per plate) and treated with PMA. After 48 h of treatment, the culture medium was discarded, and serum-free RPMI-1640 medium was added for 24 h before collection of the culture supernatant. The supernatant (100 μL) and protein standards were added to 96-well plates precoated with antibodies for detection of human TNF-α, IL-1β, MIF, CXCL5, and IL-8 by using the corresponding ELISA kits (Multi Sciences) according to the manufacturer’s protocol. The plates were finally placed in a SpectraMax i3 instrument, and the absorbance was measured at 450 nm and 630 nm for quantification. The concentrations were calculated from the standard curve and the absorbance values at 450 nm and 630 nm.

### MTT assay

Transfected macrophages (differentiated from THP-1 cells) were cultured with RPMI-1640 medium without FBS for 24 h before collection of the culture supernatants. The HNSCC cell lines HN6 and CAL27 were seeded into 96-well plates at a density of 800 cells per well with 100 μL of each culture supernatant and incubated at 37 °C. The cells were incubated with 3-(4,5-dimethylthiazol-2-yl)−2,5-diphenyltetrazolium bromide (MTT) (Yeasen) at a final concentration of 0.5 mg/mL in the medium for 4 h on day 0 to day 6. The resulting formazan crystals were solubilized by incubation with 150 μL of dimethyl sulfoxide (Sangon Biotech) for 5 min with shaking, and the absorbance of the samples was measured using a SpectraMax i3 at 570 nm.

### Colony formation assay

A colony formation assay was used to test cell proliferation. Transwell chambers were placed in a six-well plate to separate tumor cells and transfected macrophages (differentiated from THP-1 cells). HN6 and CAL27 cells were seeded in six-well plates at 1000 cells per well and incubated with macrophage supernatant until obvious clonal proliferation was observed. The colonies were fixed with 4% PFA for 15 min and stained with 0.1% v/v crystal violet for another 15 min. Images were acquired using a scanner (WIA CanoScan 5600F). The number of colonies containing more than 50 cells was quantified for further analysis.

### Cell migration: transwell and wound healing assays

For the migration assay, Transwell chambers (pore size, 0.8 mm; Corning) were placed in a 24-well plate, and the supernatants of transfected macrophages and the inducers were added to the lower compartments. Then, HN6 and CAL27 cell suspensions were added separately to the upper compartments, and the plate was incubated at 37 °C for 24 h. After the cells were allowed to migrate into the lower compartments, the Transwell chambers were removed, and the cells were fixed with 4% PFA (Biosharp) for 15 min, stained with 0.1% v/v crystal violet (Solarbio) for 15 min, and photographed.

A wound healing assay was also used to evaluate cell migration. HN6 and CAL27 cells were seeded in a six-well plate one day in advance. After culture to confluence, the cell monolayer was scraped with a P200 tip (the time of wounding was recorded as time 0). The culture medium was then discarded, and the supernatant of transfected macrophages were added to the cells for incubation. Images of 5 nonoverlapping fields were obtained after 24 h.

### Isolation of nuclear and cytoplasmic proteins from macrophages

Transfected THP-1 cells were seeded in 10-cm plates (8 × 10^6^ cells per plate) and treated with PMA for 48 h. The culture medium was discarded, and the cells were washed with ice-cold PBS 2 times. Accutase (Millipore) was used to digest the macrophages at 37 °C for 15 min. The cells were then collected by centrifugation at 1200 rpm for 5 min. Lysis buffer (10 mM PIPES (pH = 6.8), 100 mM NaCl, 300 mM sucrose, 3 mM MgCl2, 1 mM EDTA, and 0.5% v/v Triton X-100) was added to resuspend the macrophages, and after incubation on ice for 15 min, the cytoplasmic proteins were collected by centrifugation at 4000 rpm for 5 min at 4 °C. Before nuclear proteins were collected, the supernatants were discarded, and the cell pellets were resuspended in lysis buffer and subjected to 2 additional rounds of centrifugation at 4000 rpm for 5 min at 4 °C. The nuclear proteins were heated at 105 °C for 20 min. Western blot analysis was subsequently performed to detect p65 expression in the nucleus and cytoplasm of macrophages.

### Flow cytometry

HN6, CAL27 cells and macrophages were were digested into single-cell suspensions with 0.25% Trypsin–EDTA (New Cell & Molecular Biotech) and Accutase (Sigma). Then cells were incubated with LIVE/DEAD™ Fixable Violet Dead Cell Stain Kit (Thermo) for 30 min on ice. After fixation and permeabilization (BD Biosciences) for 30 min on ice, antibodies (OPN-eFluor 660, Ebioscience) were incubated in PBS for 30 min on ice. NovoCyte Peatean (Agilent) was used to analyze single-cell suspension samples.

### In vivo tumorigenicity assay

Six-week-old male BALB/c nude mice were purchased from Shanghai Bikai Keyi Biotechnology Co., Ltd. All the mice were bred and housed in the animal facility of Shanghai Ninth People’s Hospital under specific pathogen-free conditions. To evaluate the promoting role of SPP1 + Macs in vivo, subcutaneous cotransplantation models were used. HN6 cells and pretreated transfected macrophages in 10-cm dishes were washed with prechilled PBS and detached with 0.25% trypsin (Gibco) and Accutase separately. The cells were collected by centrifugation and were then resuspended in Matrigel (Corning) at a HN6 HNSCC tumor cell:macrophage ratio of 7:3 [16, 17]. Insulin syringes (KDL) were used to inject 20 μL of the cell/Matrigel mixture (a total of 2.3 × 10^6^ HN6 cells and 1 × 10^6^ macrophages) into the backs of the mice. The tumor volume was calculated as V = 1/2 × length × width^2^. The tumors were harvested after 18 days and were then weighed, photographed, fixed and embedded in paraffin for IHC and multiplex immunofluorescence analysis.

### Statistical analysis

Statistical analyses and data plotting were performed with R Statistical Software and GraphPad Prism 8. The significance of differences among more than two groups and between two groups was determined by one-way ANOVA and Student’s t test, respectively. HNSCC patients were divided into low-expression and high-expression groups according to the median SPP1 expression level. Kaplan–Meier survival analysis with the log–rank test was used to compare the survival of HNSCC patients based on stratification by gene expression level. Receiver operating characteristic (ROC) curves were used to compare tumor tissues and adjacent normal tissues. All *p* values were two-sided, and differences with *p* < 0.05 were considered statistically significant.

## Results

### Tumor-specific SPP1 + Macs were identified by scRNA-seq in HNSCC clinical samples

**Fig. 1:**
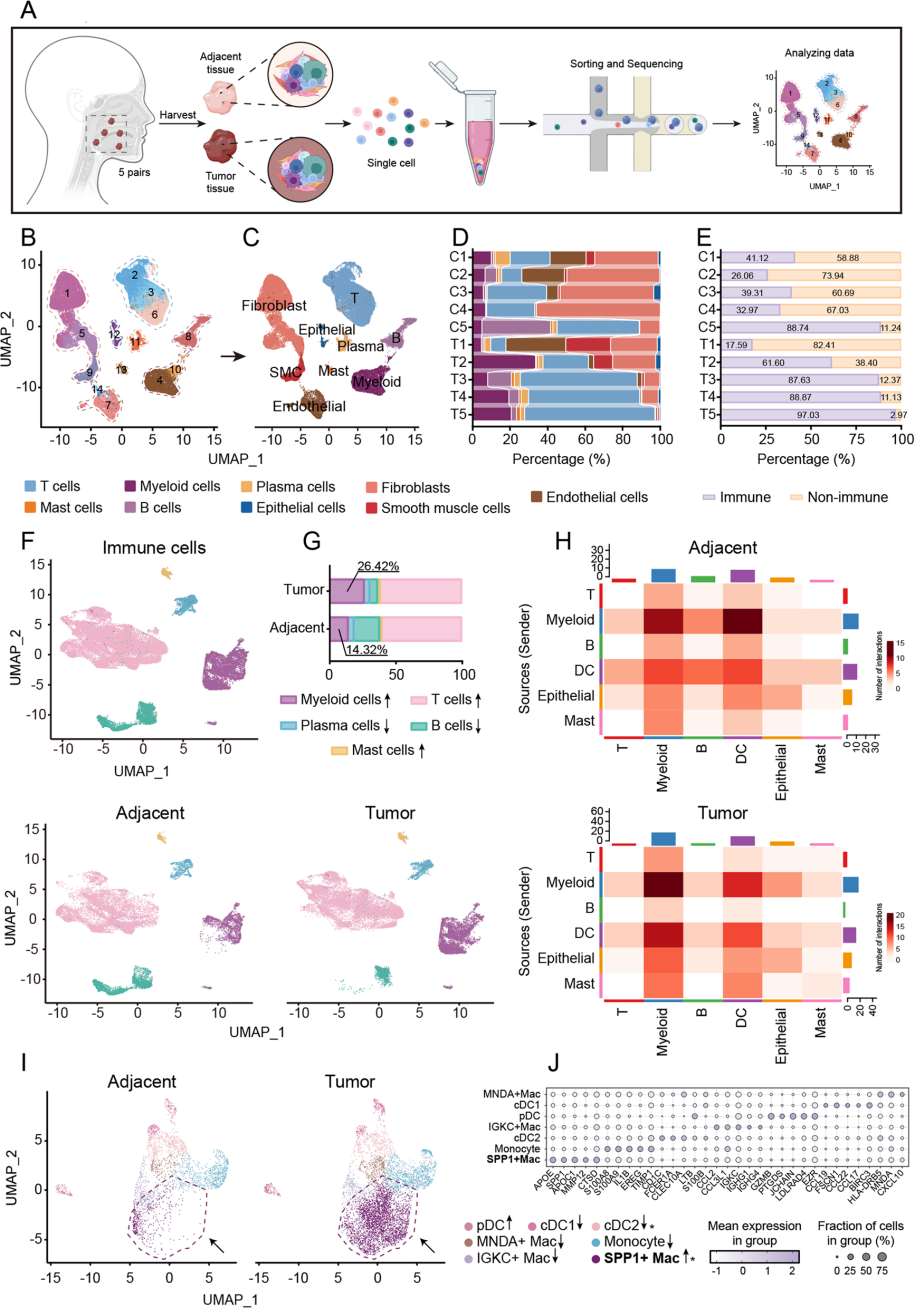
Tumor-specific SPP1^+^ macrophages in HNSCC tissues. **A** Schematic diagram of the scRNA-seq method used in this study. Adjacent normal tissues and tumor tissues from 5 HNSCC patients were processed into single-cell suspensions, and unsorted cells were subjected to scRNA-seq with the 10 × Genomics protocol. **B** UMAP plot of the 14 cell clusters. **C** UMAP plot of the 9 major cell types. The cell types were identified by marker genes. **D** Bar plots showing the percentages of the 9 major cell types in adjacent normal tissues and tumor tissues from 5 HNSCC patients. **E** Bar plots showing the proportions of immune cells and nonimmune cells in different tissues. The percentages of both cell types are shown. **F** Combined UMAP plot showing clusters of cells in adjacent normal tissues (left panel), tumor cells (right panel) and total immune cells (up panel) from 5 HNSCC patients. **G** Bar charts showing the proportions of myeloid cells among immune cells in adjacent normal tissues and tumor tissues. The percentages of myeloid cells are shown on the charts. **H** Cell interaction analysis of immune cells and epithelial cells in adjacent normal tissues (up panel) and tumor tissues (down panel). **I** UMAP plots showing the 7 clusters of myeloid cells identified in adjacent normal tissues and tumor tissues of 5 HNSCC patients. Each cluster is shown in a different color. **J** Dot plots showing the expression of known markers in myeloid cell clusters. The dot size represents the percentage of cells expressing the genes in each cluster. The expression intensity of the marker genes is shown. C, adjacent normal tissue; T, tumor tissue. *p<0.05

To determine the cellular composition of HNSCC, 5 pairs of adjacent normal tissues and tumor tissues were collected and digested into single-cell suspensions for scRNA-seq analysis (Fig. 1A). After quality control and cell doublet filtration, the transcriptomes of 71,539 cells (39,202 cells from adjacent normal tissues and 32,337 cells from tumor tissues) were retained for subsequent analysis. The cells were divided into 14 clusters through dimensionality reduction via uniform manifold approximation and projection (UMAP) and then classified into nine major cell types (Fig. 1B, C) as follows: epithelial cells (*n* = 855), fibroblasts (*n* = 21,769), smooth muscle cells (*n* = 2918), endothelial cells (*n* = 5413), mast cells (*n* = 715), myeloid cells (*n* = 8540), plasma cells (*n* = 1768) and T cells (*n* = 24,784) (Supplementary Fig. S1A). All nine major cell types were present in both the adjacent normal tissues and the tumor tissues of all five patients. However, the degree of infiltration of each cell type was different, which might reflect differences in the stage of HNSCC (Fig. 1D; Supplementary Fig. S1B; Supplementary Table S5). To further explore the TIME of HNSCC, we compared the proportions of immune cells and nonimmune cells between adjacent normal tissues and tumor tissues and divided the immune cells into five subsets: B cells (*MS4A1* and *BANK1*), mast cells (*TPSAB1* and *CPA3*), plasma cells (*JCHAIN* and *TNFRSF17*), myeloid cells (*FPR3* and *MS4A4A*) and T cells (*CD3D* and *CD3E*) (Fig. 1E, F; Supplementary Fig. S1C-G).

Compared the adjacent normal tissues and tumor tissues from each patient, myeloid cells, T cells and mast cells were upregulated in tumor tissues. Myeloid cells accounted for approximately 14.32% of the immune cells in all adjacent normal tissues, but their proportions differed among the patients’ tumor tissues, where they averagely accounted for 26.42% of the immune cells (Fig. 1G). The percentage of T cells showed little increase compared with myeloid cells, while the percentage of mast cells was too low. The increase in myeloid cells in tumor tissues indicated that these cells might be related to tumor progression in HNSCC. CellChat was then used to explore the communication between immune cells and epithelial cells. Compared with those in adjacent normal tissues, the interactions between myeloid cells and epithelial cells were markedly different in tumor tissues (Fig. 1H). We thus focused on myeloid cells and classified these cells into seven subclusters according to a previous study, with cDC1s, cDC2s, pDCs and monocytes characterized by high expression of *BIRC3*, *CD1C*, *GZMB* and *S100A8*, respectively. Three distinct subsets of macrophages—SPP1^+^ Macs, MNDA^+^ Macs and IGKC^+^ Macs—were also identified (Fig. 1I, J). Interestingly, both MNDA^+^ macrophages and IGKC^+^ macrophages were enriched in adjacent normal samples, whereas SPP1^+^ Macs were increased in almost all tumor samples (Fig. 1I). These results reveal that SPP1^+^ Macs may perform distinct functions in the malignant progression of HNSCC.

### Tumor-specific SPP1 + Macs are positively associated with poor HNSCC prognosis

**Fig. 2:**
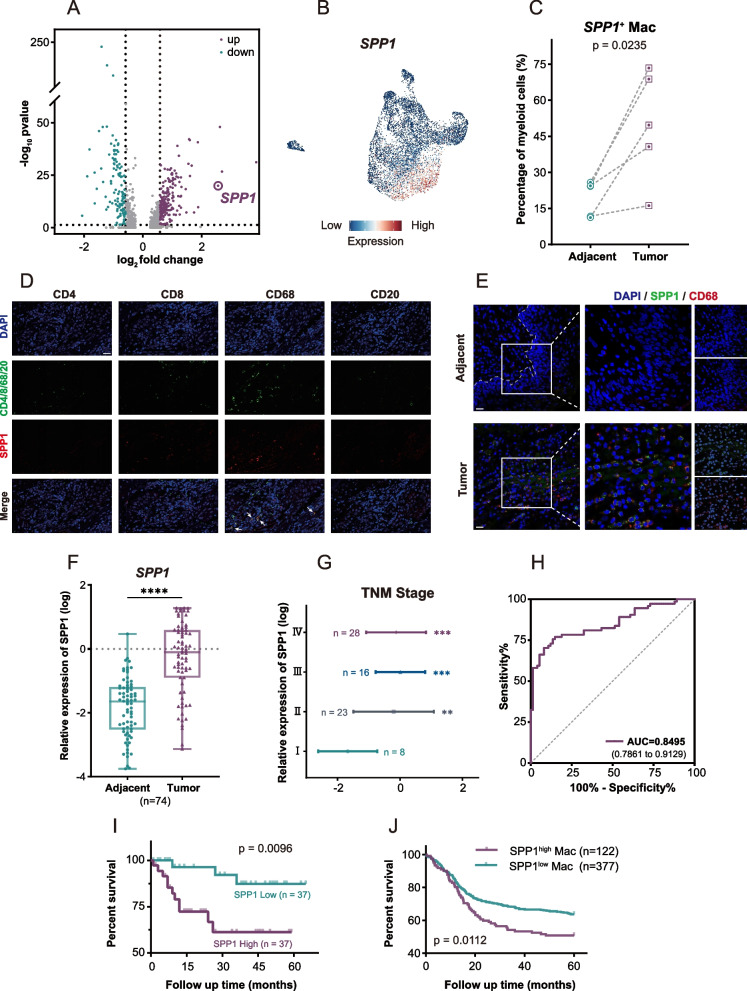
The presence of SPP1^+^ Macs is positively associated with poor prognosis in HNSCC. **A** Volcano plot showing the upregulated genes and downregulated genes in myeloid cells. Adjusted *p* value < 0.05. **B** UMAP plots of all myeloid cells colored according to the expression level of SPP1. **C** Comparison of SPP1^+^ Mac percentages in paired adjacent normal tissues (*n* = 5) and tumor tissues (*n* = 5). **D** Representative IF staining of SPP1 (red) and various immune-associated cell types (green), including macrophages (CD68 +), CD4 + T cells, CD8 + T cells and B cells (CD20 +), and DAPI staining (blue) in human HNSCC tissues. Scale bar, 20 μm. **E** Representative IF staining of SPP1 (red) and CD68 (green) in paired adjacent tissues and HNSCC tissues. Scale bar, 20 μm. **F** PCR analysis of SPP1 expression in paired normal tissues (*n* = 74) and HNSCC tissues (*n* = 74). **G** Upregulated SPP1 expression compared with that in patients with stage I disease was correlated with advanced tumor stage. **H** ROC curves showing the ability of SPP1 to distinguish HNSCC tissues from adjacent normal tissues. **I** Kaplan‒Meier analysis of overall survival based on the SPP1 expression level in 74 pairs of HNSCC tissues. **J** Overall survival rate according to the SPP1^+^ Mac expression level in HNSCC tissues in a TCGA cohort. (**p < 0.01; ***p < 0.001; ****p < 0.0001)

Since SPP1^+^ Macs are almost universally present in tumor tissues, we next investigated SPP1 expression in myeloid cells. The volcano plots and UMAP plots revealed that SPP1 was significantly upregulated in myeloid cells and highly expressed in macrophages (Fig. 2A, B). Moreover, SPP1^+^ Macs accounted for 49.77% of the total myeloid cell population in tumor tissues but only 19.52% of the total myeloid cell population in adjacent normal tissues (Fig. 2C). MIHC staining revealed that SPP1 was substantially colocalized with CD68^+^ cells but barely colocalized with CD4^+^ T cells, CD8^+^ T cells or B cells and that SPP1 was more highly expressed in macrophages derived from HNSCC tumor tissues compared with those derived from adjacent normal tissues (Fig. 2D, E, Supplementary Fig. S2D). Real-time PCR was then used to measure the SPP1 expression level in 74 pairs of HNSCC tissue samples and adjacent normal oral epithelial tissue samples. SPP1 expression was significantly greater in HNSCC tumor samples than in normal oral epithelial tissue samples (Fig. 2F). Similarly, analysis of The Cancer Genome Atlas (TCGA) database revealed that SPP1 was highly expressed in HNSCC (Supplementary Fig. S2A). Further analysis revealed that SPP1 expression was positively correlated with TNM stage (Fig. 2G). In addition, the area under the curve (AUC) for SPP1 was 0.8495 (95% CI, 0.7861–0.9129), which indicated that SPP1 might be a biomarker for HNSCC (Fig. 2H). Indeed, patients with higher SPP1 expression exhibited a lower survival rate (Fig. 2I). Analysis of TCGA data revealed consistent findings, which suggested that SPP1^+^ Macs were associated with poor outcomes and that the SPP1 expression level was positively correlated with cancer stage and tumor grade in HNSCC patients (Fig. 2J; Supplementary Fig. S2B, C). Collectively, these results indicate that SPP1 is upregulated in HNSCC and is expressed predominantly in macrophages, which suggests the potential value of SPP1 as a prognostic biomarker.

### SPP1 + Macs promote HNSCC progression by secreting cytokines

**Fig. 3:**
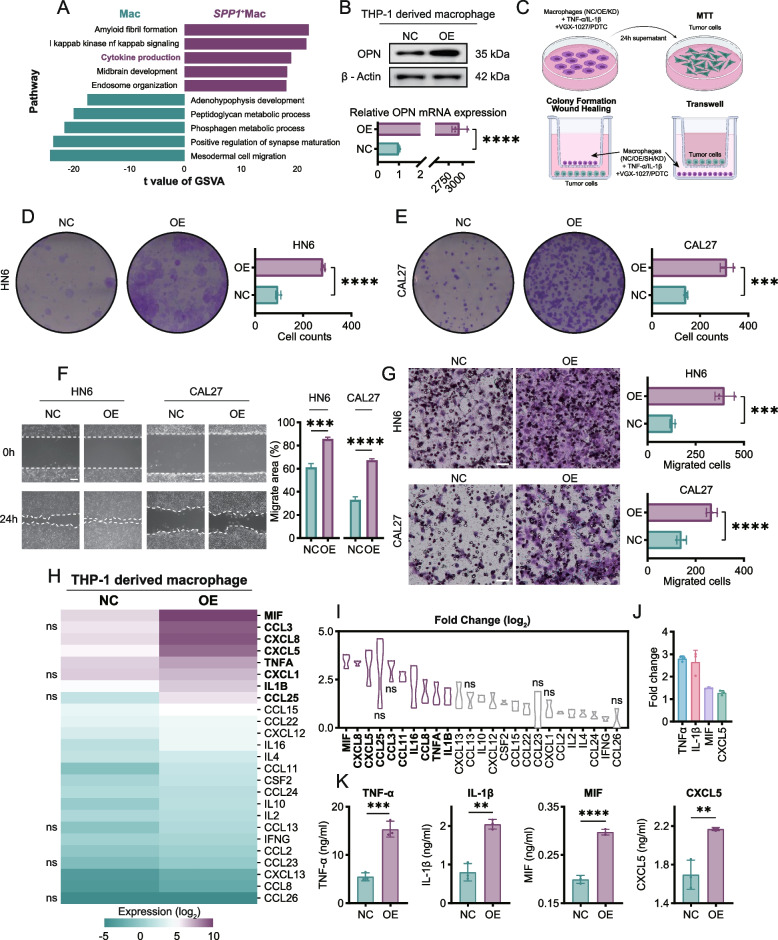
SPP1^+^ Macs promote HNSCC progression by secreting cytokines. **A** Differential pathway enrichment in all other Mac and SPP1^+^Mac (scRNA-seq) as determined by GSVA. Two-sided unpaired limma moderated t test. **B** Immunoblot analysis and real-time qPCR analysis were used to measure SPP1 protein and mRNA levels, in SPP1-OE macrophages, respectively. **C** Schematic diagram of tumor cell culture in macrophage supernatant or coculture with macrophages for in vitro experiments. **D**, **E** The colony-forming ability of HN6 (**D**) and CAL27 (**E**) cells was increased when they were cocultured with SPP1-OE macrophages. **F**, **G** The migration ability of HN6 and CAL27 cells was increased by coculture with SPP1-OE macrophages, as determined using wound healing assays (**F**) and Transwell assays (**G**). The data are presented as the mean ± SD of three independent experiments. Scale bar, 50 μm. **H**, **I** Luminex liquid suspension chip detection assay of SPP1-NC and SPP1-OE macrophages (derived from THP-1 cells). The expression intensity of each cytokine is shown. **J**, **K** Secretion of the indicated proteins, as determined by ELISA. TNF-α, IL-1β, MIF, CXCL5 were the top 5 cytokines with significant fold change and expression and verfied by ELISA. (ns, no significant difference; *p < 0.05; **p < 0.01; ***p < 0.001; ****p < 0.0001). GSVA, gene set variation analysis. Macs, macrophages. NC, THP-1 cell-derived SPP1-NC macrophages. OE, THP-1 cell-derived SPP1-OE macrophages

To assess the biological effect of SPP1^+^ Macs on HNSCC, we divided macrophages into two subsets, SPP1^+^ Macs and all other macrophages, and performed gene set variation analysis (GSVA) based on the scRNA-seq data. The results indicated that SPP1^+^ Macs were actively involved in cytokine production compared with macrophages (Fig. 3A). Thus, we hypothesized that SPP1^+^ Macs might promote HNSCC progression through cytokine secretion. Here, we utilized lentiviral transduction to overexpress or knock down SPP1 in THP-1 cells. After the THP-1 cells were induced to differentiate into macrophages by treatment with PMA for 48 h, the protein and mRNA levels of SPP1 in THP-1 cell-derived macrophages were then measured by western blotting and real-time PCR, respectively (Fig. 3B; Supplementary Fig. S3B). To investigate the direct or indirect effects of SPP1 + Macs on HNSCC cells, cell culture supernatants from the SPP1-negative control (SPP1-NC; SPP1-NC THP-1 cell-derived macrophages) group and the SPP1-overexpressing (SPP1-OE; SPP1-OE THP-1 cell-derived macrophages) group were collected for further indirect culture with HN6 and CAL27 cells, while another coculture model was used to explore the direct effects (Fig. 3C). Compared with SPP1-NC, SPP1-OE promoted the proliferation of HN6 and CAL27 cells in both the direct and indirect culture systems, while the direct coculture system strongly accelerated HNSCC cell growth (Fig. 3D, E; Supplementary Fig. S3A). Since SPP1 has been reported to promote tumor cell migration [18], wound healing and Transwell migration assays were performed to explore whether SPP1 + Macs exerted a similar effect on HNSCC tumor cells. Notably, SPP1-OE strongly promoted the migration of HN6 and CAL27 cells (Fig. 3F, G). Additionally, SPP1 short hairpin RNA negative control (SPP1-Sh-NC; SPP1-Sh-NC THP-1 cell-derived macrophages) and SPP1-knockdown (SPP1-KD; SPP1-KD THP-1 cell-derived macrophages) cells were also cocultured with HN6 and CAL27 cells. Compared with SPP1-Sh-NC, coculture with SPP1-KD decreased the proliferation and migration abilities of these cells (Supplementary Fig. S3C-F).

To further explore the mechanism by which SPP1 + Macs release candidate cytokines that act on HNSCC cells, Luminex liquid suspension chip detection was used to compare the expression of 40 common chemotactic and inflammatory cytokines between the SPP1-NC and SPP1-OE groups. MIF, CXCL8, CXCL5, TNF-α and IL-1β were the 5 most highly differentially expressed cytokines in the SPP1-OE group compared with those in the SPP1-NC group (Fig. 3H, I). ELISA was then used to measure the concentrations of these cytokines in the fresh supernatants of the SPP1-NC and SPP1-OE cell groups. The results indicated that MIF, TNF-α and IL-1β were the most significantly upregulated and highly expressed genes in SPP1-OE macrophages (Fig. 3J, K; Supplementary Fig. S3G). Therefore, we focused on these three factors in subsequent experiments.

### SPP1 + Mac-derived TNF-α and IL-1β accelerate the proliferation and migration of HNSCC cells in vitro

To further explore the role of SPP1 + Mac-derived MIF, TNF-α and IL-1β, exogenous rhMIF, rhTNF-α and rhIL-1β were each added separately to the supernatant of SPP1-NC macrophages for indirect culture with HN6 and CAL27 cells, the results of which were compared with those of the coculture with untreated supernatant from SPP1-NC macrophages. rhTNF-α and rhIL-1β, but not rhMIF, promoted HNSCC cell proliferation (Supplementary Fig. S4A-D). These data suggested that TNF-α and IL-1β might play major roles in the effects of SPP1 + Mac-derived cytokines on HNSCC cells. We also detected protein and mRNA expression of TNF-α and IL-1β in SPP1-NC, SPP1-OE, SPP1-Sh-NC and SPP1-KD. The results indicated that the endogenous expression of TNF-α and IL-1β were also changed in these macrophages (Supplementary Fig. S3H-I).

**Fig. 4:**
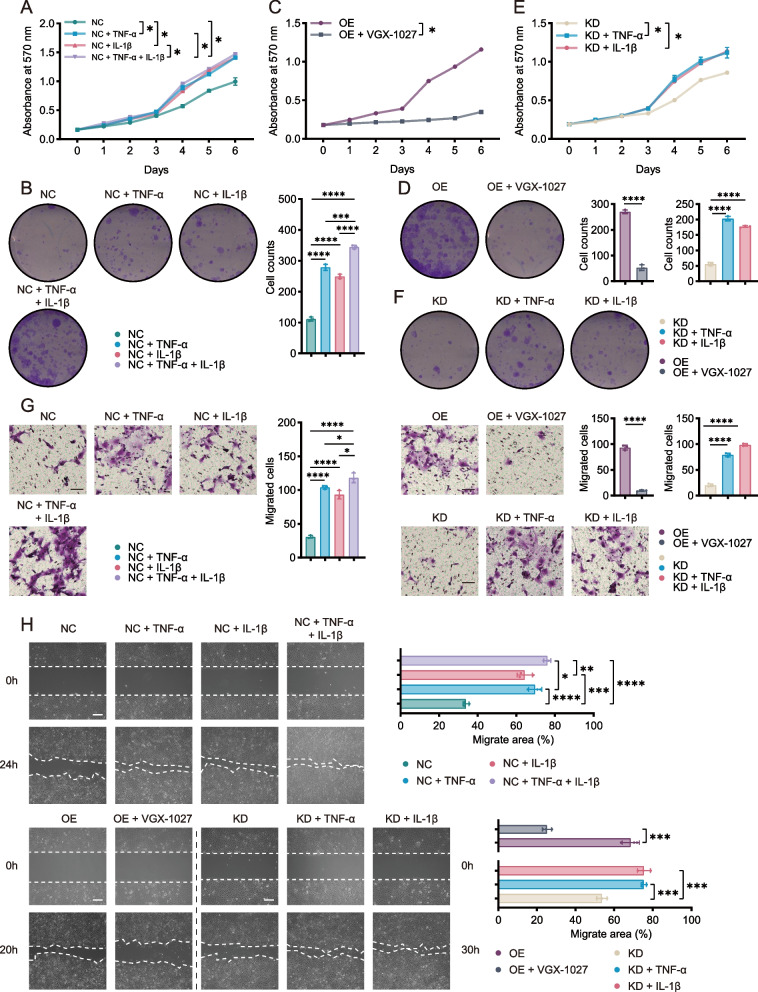
TNF-α and IL-1β produced by SPP1^+^ Macs contribute to HN6 cells proliferation and migration. **A**-**H** Growth curves and colony formation and migration abilities of HN6 cells in the SPP1-NC (NC, NC + TNF-α, NC + IL-1β, NC + TNF-α + IL-1β), SPP1-OE (OE, OE + VGX-1027) and SPP1-KD (KD, KD + TNF-α, KD + IL-1β) groups. Each group was analyzed using MTT assays (**A**, **C**, **E**), colony formation assays (**B**, **D**, **F**), Transwell assays (**G**) and wound healing assays (**H**). Scale bar, 50 μm. (*p < 0.05; **p < 0.01; ***p < 0.001; ****p < 0.0001). NC, THP-1 cell-derived SPP1-NC macrophages. OE, THP-1 cell-derived SPP1-OE macrophages. KD, THP-1 cell-derived SPP1-KD macrophages

To investigate whether SPP1 + Mac-derived TNF-α and IL-1β promote HNSCC cell proliferation, the tumor cells were treated with macrophage supernatant added TNF-α and IL-1β, or VGX-1027 [19, 20], which targets macrophages and reduces the production of TNF-α and IL-1β so as to mimic and compare the effects on tumor cells caused by macrophages. MTT assays and coculture colony formation assays revealed that, compared with the SPP1-NC cell group, the SPP1-NC + rhTNF-α, SPP1-NC + rhIL-1β and SPP1-NC + rhTNF-α + rhIL-1β cell groups induced markedly increased proliferation of HN6 and CAL27 cells (Fig. 4A, B; Supplementary Fig. S5A, B). VGX-1027 was then added to the supernatant from the SPP1-NC and SPP1-OE group to block the effects of TNF-α and IL-1β, and we found that the proliferation rate decreased dramatically in SPP1-OE group and slightly in SPP1-NC group (Fig. 4C, D; Supplementary Fig. S5C, D; Supplementary Fig. S6A-D). The results showed that inhibiting macrophage-derived TNF-α and IL-1β decreased the growth of tumor cells and SPP1-NC also exhibited the secretion of TNF-α and IL-1β. Similarly, rhTNF-α and rhIL-1β promoted HNSCC cell growth when cultured with SPP1-KD cells (Fig. 4E, F; Supplementary Fig. S5E, F), which indicated that the functions of TNF-α and IL-1β differed from those of SPP1 in SPP1 + Macs. We also added VGX-1027 to tumor cells simply, the results showed that VGX-1027 did not affect tumor cells (Supplementary Fig. S6I-L). Since SPP1 + Mac-derived TNF-α and IL-1β played important roles in HNSCC progression, we sought to determine whether these cytokines were involved in HN6 and CAL27 cell migration. Wound healing and Transwell migration assays revealed that SPP1-NC + rhTNF-α, SPP1-NC + rhIL-1β, SPP1 + rhTNF-α + rhIL-1β, SPP1-KD + rhTNF-α or SPP1-KD + rhIL-1β treatment strongly accelerated the migration of these cells, whereas VGX-1027 treatment considerably slowed the migration of HNSCC cells (Fig. 4G, H; Supplementary Fig. S5G, H). And, as positive and negative control, we also used anti-TNF-α and anti-IL-1β in SPP1-OE supernatant to verify the function of VGX-1027 and TNF-α and IL-1β in macrophages. Anti-TNF-α and anti-IL-1β in SPP1-OE supernatant had similar effect on tumor cells compared with VGX-1027 (Supplementary Fig. S6E-H, S6M-P, S7A-H). Together, these results suggest that TNF-α and IL-1β may have major roles in how SPP1 + Macs worked on promoting HNSCC cell proliferation and migration in vitro.

### TNF-α and IL-1β secretion by SPP1 + Macs is regulated by the NF-κB signaling pathway

After establishing a connection between SPP1 + Macs and TNF-α and IL-1β, we next investigated the mechanism of their secretion. Many studies have reported that OPN (encoded by SPP1) is related to activation of the NF-kappa B signaling pathway in several types of tumors, such as melanoma and renal cell carcinoma [21, 22]. GSVA of our scRNA-seq data also revealed that SPP1 + Macs were associated with the I kappa B/kinase nf-kappa B signaling pathway (Fig. 3A). Thus, we investigated whether the NF-kappa B signaling pathway influenced the secretion of TNF-α and IL-1β by SPP1 + Macs.

**Fig. 5:**
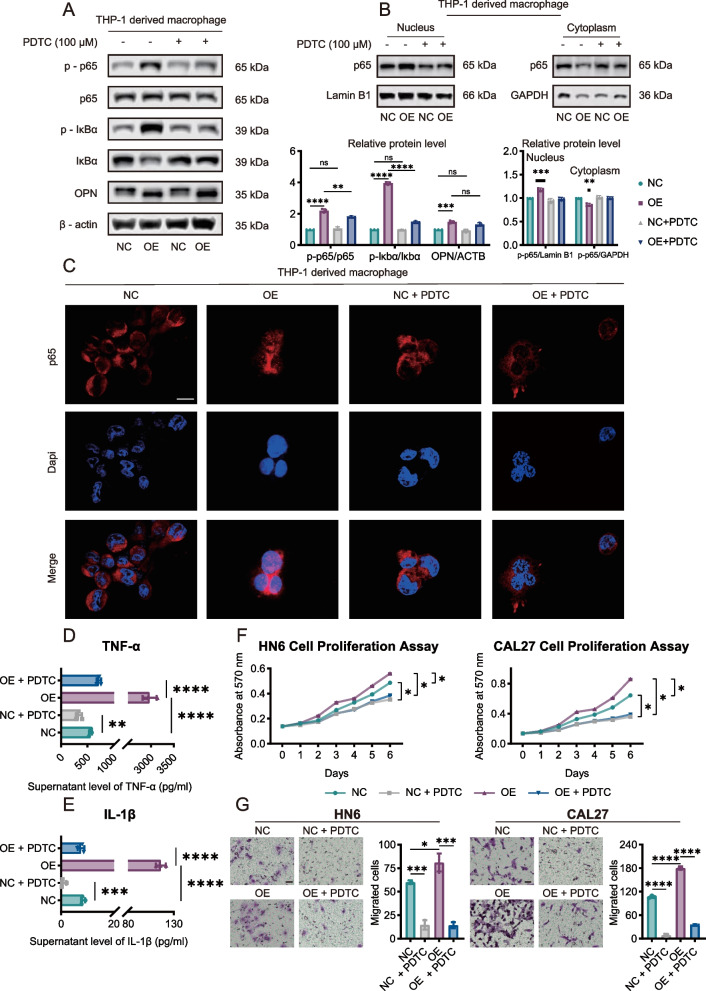
SPP1^+^ Macs promote the secretion of TNF-α and IL-1β through the NF-kappa B axis. **A** Western blot analysis of p65 expression and phosphorylation p65, IκBα and phosphorylation IκBα, and OPN expression in the NC and OE groups with or without PDTC pretreatment (100 μM) for one hour. **B**, **C** NC and OE groups were pretreated with or without 100 μM PDTC for one hour. Western blot analysis and relative protein level (**B**) and immunofluorescence assays (**C**) were used to assess the location of p65 in macrophages. Scale bar, 20 μm. **D**, **E** ELISA results showing the concentrations of secreted TNF-α and IL-1β in the NC, OE, NC + PDTC, and OE + PDTC group supernatants. **F**, **G** NC and OE groups were pretreated with or without 100 μM PDTC for one hour. HN6 and CAL27 cells were cultured with the supernatants from these cells or cocultured with the cells themselves. Cell proliferation (**F**) and Transwell migration (**G**) assays were performed to analyze alterations in tumor cell growth and migration in the different groups. Scale bar, 50 μm. (*p < 0.05; **p < 0.01; ***p < 0.001; ****p < 0.0001). NC, THP-1 cell-derived SPP1-NC macrophages. OE, THP-1 cell-derived SPP1-OE macrophages. KD, THP-1 cell-derived SPP1-KD macrophages

We pretreated SPP1-NC and SPP1-OE with PDTC, a selective NF-kappa B inhibitor [23] for 3 h to assess the effect of SPP1-OE macrophage. Compared with SPP1-NC macrophages, SPP1-OE macrophages presented increased phosphorylation of p65 and IκBα and decreased expression of IκBα. The PDTC treatment attenuated the phosphorylation of p65 and IκBα (Fig. 5A). We also separated the nuclear and cytoplasmic fractions from macrophages to detect p65 translocation by western blotting (Supplementary Fig. S7I). p65 was localized mainly in the cytoplasm in SPP1-NC macrophages, whereas in SPP1-OE macrophages, p65 accumulated in the nucleus. After pretreatment with PDTC, p65 remained in the cytoplasm (Fig. 5B). The results of confocal microscopy also confirmed these results and indicated that the NF‒kappa B signaling pathway may be activated in SPP1 + Macs (Fig. 5C). ELISA was subsequently used to determine whether the inhibition of NF-kappa B signaling in macrophages by PDTC decreased the secretion of TNF-α and IL-1β. PDTC dramatically reduced the secretion of TNF-α and IL-1β from both SPP1-NC and SPP1-OE macrophages (Fig. 5D, E). Moreover, MTT and Transwell migration assays revealed that pretreatment of macrophages with PDTC suppressed the proliferation and migration of HN6 and CAL27 cells upon coculture with these macrophages (Fig. 5F, G). In summary, our findings demonstrate that SPP1 + Macs increase the secretion of TNF-α and IL-1β by activating the NF-kappa B signaling pathway.

### SPP1 + Mac-derived TNF-α and IL-1β promote the expression of OPN in both macrophages and tumor cells

**Fig. 6:**
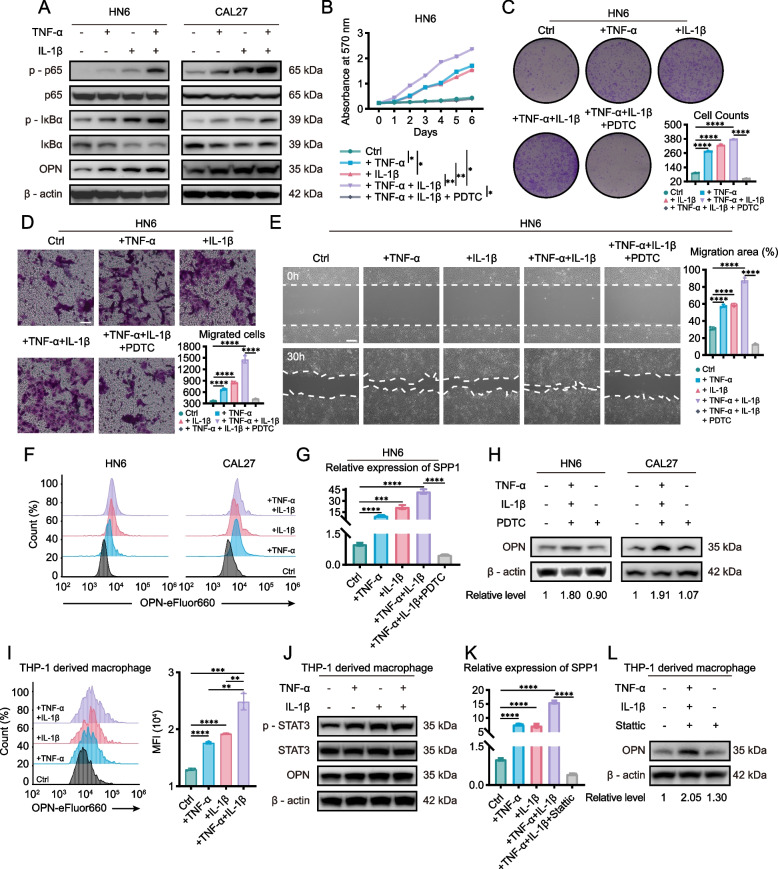
TNF-α and IL-1β promote the expression of OPN in tumor cells and macrophages. **A** TNF-α and IL-1β activated NF-kappa B signaling pathway in tumor cells. **B**-**E** Growth curves and colony formation and migration abilities of HN6 cells stimulated by TNF-α, IL-1β and NF-kappa B inhibitor, PDTC. MTT assays (**B**), colony formation assays (**C**), Transwell assays (**D**) and wound healing assays (**E**) were performed. Scale bar, 50 μm. **F** Flow Cytometry showed that TNF-α and IL-1β promoted the expression of OPN in tumor cells. **G** PCR was used to measure the fold change of OPN in mRNA level in HN6. **H** PDTC inhibited OPN expression in tumor cells. Relative level was calculated by ImageJ. **I** TNF-α and IL-1β promoted the expression of OPN in macrophages, which was determined by FCS. **J** TNF-α and IL-1β activated STAT3 to increase the expression of OPN in macrophages. **K**, **L** STAT3 inhibitor, Stattic, inhibited the elevated level of OPN caused by TNF-α and IL-1β in macrophages, both in mRNA (**K**) and protein (**L**) level. (*p < 0.05; **p < 0.01; ***p < 0.001; ****p < 0.0001)

To further explore how TNF-α and IL-1β secreted from SPP1^+^ Mac cells promote the progression of HNSCC, rhTNF-α and rhIL-1β were added to HN6 and CAL27 tumor cells both individually and together. Previous studies have shown that TNF-α and IL-1β activate the PI3K/Akt, NF-κB, MAPK/Erk and STAT3 signaling pathways in different cell types [23–26]. Thus, we detected which signaling pathway was associated with the ability of TNF-α and IL-1β to regulate tumor cells. Western blot analysis revealed that in both HN6 and CAL27 cells, the NF-κB signaling pathway was activated by TNF-α and IL-1β alone and by both TNF-α and IL-1β together (Fig. 6A; Supplementary Fig. S8A-E). Moreover, PDTC decreased the TNF-α- and IL-1β-induced increase in the proliferation and migration abilities of tumor cells (Fig. 6B-E; Supplementary Fig. S8F-I). Interestingly, we also observed that OPN expression in tumor cells was also increased when they were treated with TNF-α or IL-1β, and a dual effect was shown when they were treated with both TNF-α and IL-1β (Fig. 6A, F-G; Supplementary Fig. S8J, K). We further used flow cytometry to detect the expression and PDTC to inhibit the function (Fig. 6G, H; Supplementary Fig. S8J, K). The results showed that TNF-α and IL-1β activated the NF-κB signaling pathway and then promoted OPN expression in tumor cells and tumor cell progression. Given that TNF-α and IL-1β promoted the expression of OPN in tumor cells, we wondered whether TNF-α and IL-1β promote the expression of OPN in adjacent macrophages. When macrophages were treated with TNF-α or IL-1β alone or with both TNF-α and IL-1β, OPN expression was upregulated at both the protein and mRNA levels (Fig. 6I). However, we found that TNF-α and IL-1β activated STAT3 but not the NF-κB signaling pathway in macrophages. Using Stattic, which block STAT3, in macrophages with TNF-α and IL-1β decreased OPN expression (Fig. 6J-L; Supplementary Fig. S8L-O). Briefly, SPP1 + Mac-derived TNF-α and IL-1β promote the expression of OPN in both macrophages and tumor cells.

### SPP1 + Macs promote HNSCC progression in vivo

**Fig. 7:**
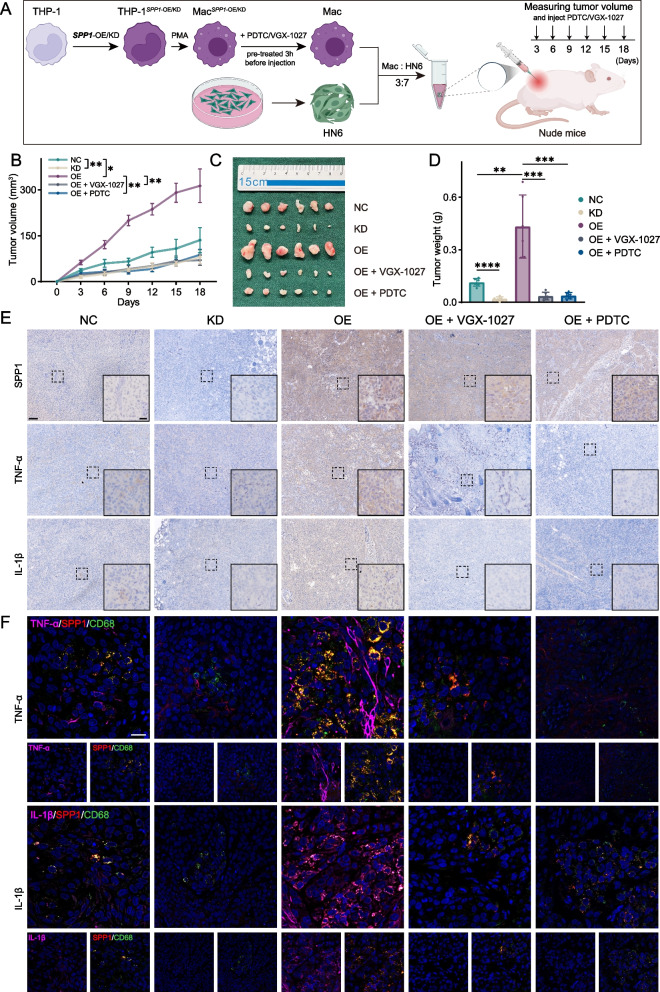
Effects of SPP1^+^ Macs on promoting tumor growth in vivo. **A** Schematic diagram showing the process for establishing the subcutaneous tumor models by coinjection of HN6 cells and NC, OE, KD, OE + VGX-1027 and OE + PDTC macrophages. Tumors were harvested 18 days after cells injection. **B** Tumor growth after subcutaneous coinjection of HN6 cells and macrophages. **C** Images of isolated tumors. **D** Tumor weight at 18 days post-injection with HN6 cells and NC, OE, KD, OE + VGX-1027, or OE + PDTC macrophages. **E** IHC analysis of SPP1, TNF-α, and IL-1β expression in the tumors from different groups. Scale bars: left: 100 μm; right: 50 μm. **F** Representative mIHC staining of SPP1 (red), a macrophage marker (CD68 + , green), and cytokines (TNF-α and IL-1β, violet) and DAPI staining (blue) in mouse tumor tissues. Scale bar, 20 μm. NC, THP-1 cell-derived SPP1-NC macrophages. OE, THP-1 cell-derived SPP1-OE macrophages. KD, THP-1 cell-derived SPP1-KD macrophages. (*p < 0.05; **p < 0.01; ***p < 0.001; ****p < 0.0001)

To further verify the effect of SPP1 + Macs on HNSCC growth in vivo, a 7:3 mixture of HN6 HNSCC tumor cells and macrophages (differentiated from THP-1 cells) was used to establish a mouse model. After treatment with VGX-1027 or PDTC for 3 h, the SPP1-NC, SPP1-OE, SPP1-KD, SPP1-OE + VGX-1027 or SPP1-OE + PDTC cell groups were subcutaneously injected into nude mice (Fig. 7A). To keep the long-lasting effect, we injected VGX-1027 and PDTC every 3 day around the tumor. Interestingly, the tumor weights and tumor volumes were significantly lower in the SPP1-KD, SPP1-OE + VGX-1027 and SPP1-OE + PDTC groups than in the SPP1-NC group (Fig. 7B-D). After the xenograft tumors were harvested, IHC staining and mIHC staining were performed to explore the expression levels of OPN, TNF-α and IL-1β. The results of both staining assays indicated that OPN expression was greater in the SPP1-OE, SPP1-OE + VGX-1027 and SPP1-OE + PDTC groups than in the other groups. We also found that the OPN expression in SPP1-OE + PDTC showed less than the other two groups, which may indicate that PDTC inhibited the expression of OPN in tumor cells as we shown before. The TNF-α and IL-1β expression was lower in the SPP1-OE + VGX-1027 and SPP1-OE + PDTC groups than in the SPP1-OE group and was even lower than that in the SPP1-NC group, and additionally, the results showed that the expression of OPN, TNF-α and IL-1β was the lowest in the SPP1-KD group (Fig. 7E, F). In conclusion, these data demonstrate that SPP1 + Macs promote HNSCC progression by secreting the cytokines TNF-α and IL-1β via activation of the NF-kappa B signaling pathway, which is consistent with the in vitro results*.*

## Discussion

The TIME is a distinctive milieu that contains many cell types and a multitude of factors that affect tumor cells [24, 25]. As pivotal players in the TIME, myeloid cells exhibit substantial heterogeneity among HNSCC patients. Among myeloid cells, macrophages constitute the predominant subpopulation and play functional roles in tumor development, metastasis and therapeutic responses. According to their phenotype and function, macrophages can be subdivided into two major subtypes: proinflammatory M1-like macrophages and anti-inflammatory M2-like macrophages [26, 27]. However, the coexpression of M1-like and M2-like signatures within the same macrophage subpopulation has recently been identified in almost all cancer types [7, 28]. Thus, a better understanding of the crucial macrophage subpopulations in the TIME and their impacts on tumor progression is needed. Here, we applied scRNA-seq to determine the differences in myeloid cells between adjacent normal tissues and tumor tissues from HNSCC patients and identified SPP1 + Macs as a crucial subtype of macrophages in HNSCC. The population of SPP1 + Macs was dramatically increased in tumor tissue-derived myeloid cells. In addition, we verified the presence of SPP1 + Macs in clinical samples and further elucidated the effect of SPP1 + Macs on HNSCC tumor cells.

SPP1 + Macs, with SPP1 expression as one of their salient characteristics, correspond to the SPP1 + macrophages described by Zhang et al. in CRC [29]. An extensive analysis including various cancer types revealed that half of all cancers displayed SPP1-expressing clusters of macrophages and that SPP1 + Macs were associated with a worse prognosis [30–33]. By utilizing scRNA-seq and spatial transcriptomics data, macrophages can be divided into distinct functional subtypes. According to recent studies, SPP1 + Macs function as Angio-TAMs or Inflam-TAMs, which promote malignant tumor progression in diverse cancer types [6, 34]. Moreover, a pancancer study across 8 tumor types revealed that SPP1 + Macs presented the strongest M2 signature, whereas another study in CRC revealed that SPP1 + Macs also exhibited high M1 macrophage regulatory activity [34, 35]. Thus, SPP1 + Macs are universally represented in different cancers and exhibit functional diversity. In HNSCC, the expression of the macrophage polarity marker CXCL9-SPP1 (CS), but not M1 or M2 markers, is strongly negatively correlated with prognosis and can be used to identify protumor and antitumor pathways in the TME [6]. Another study focused on HNSCC revealed that SPP1 + TAMs might enhance tumor intravasation and metastasis via the secretion of SPP1, CCL18, and CXCL8 [36]. Previous studies focused on SPP1 + Macs were conducted mainly using bioinformatic analyses. The function of SPP1 + Macs in interactions with tumor cells in HNSCC has not been fully characterized. In this study, we identified a different regulatory way by which SPP1 + Macs released the cytokines TNF-α and IL-1β to promote tumor progression. Moreover, SPP1 + Mac-derived TNF-α and IL-1β can promote the expression of OPN in both tumor cells and adjacent normal macrophages in turn, which may transform into SPP1 + Macs. Inhibiting SPP1 + Mac-derived TNF-α and IL-1β suppressed tumor growth both in vivo and in vitro.

SPP1, also known as OPN, functions as a crucial adhesion protein and plays a major role in numerous tumors [8, 37, 38]. It is widely acknowledged that the tumor-promoting role of SPP1 is performed through the modulation of cancer-related signaling pathways or the TME. For example, studies on CRC have shown that OPN increases the migration and invasiveness of CRC cells by activating the PI3K/AKT pathway [39, 40]. In addition, in patients with HCC, OPN induces activation of the PI3K/AKT signaling pathway to promote EMT and metastasis and promotes angiogenesis through NF-kappa B signaling [41, 42]. OPN is also expressed in diverse cells in the TME, including cancer cells, NK cells, T cells and macrophages [43]. Previous studies have shown that OPN is upregulated in tumor cells and that elevated OPN expression promotes tumor cell proliferation, migration and invasion [18, 44]. In our study, SPP1 was found to be highly expressed in myeloid cells in tumor tissues, especially in TAMs. Consistent with the oncogenic functions of OPN in various cancers, endogenous OPN in macrophages also contribute to HNSCC cell progression and migration. Knockdown of SPP1 in macrophages inhibited growth and reduced the migration of HNSCC cells. In line with the results of the cell experiments, the results of our subcutaneous coinjection tumor model in nude mice confirmed the role of SPP1 in macrophages in tumorigenesis and the suppressive effect of SPP1 knockdown in macrophages on tumors.

TNF-α, a member of the TNF/TNFR cytokine superfamily, is involved in inflammation and is secreted predominantly by macrophages. TNF-α plays a critical role in the pathobiology of cancer, as it functions either as a protumor cytokine that favors cell proliferation or as an antitumorigenic component in different types of cancers. In HNSCC, TNF-α plays a key role in promoting angiogenesis, metastasis and tumor progression [45–47]. IL-1β is induced by inflammatory signals in immune cells, and sustained IL-1β production may promote both tumor induction and subsequent tumor propagation [48, 49]. Recent studies have shown that IL-1β is a marker of Inflam-TAMs, and in PDAC, IL-1β^+^ TAMs promote tumor growth after activation via the PGE2‒IL-1β axis [50]. IL-1β secreted by macrophages has been reported to promote drug resistance in HNSCC [51]. Luminex liquid suspension chip detection revealed that compared with macrophages, SPP1 + Macs expressed significantly higher levels of both TNF-α and IL-1β. Pretreatment of either macrophages or SPP1-KD macrophages with TNF-α and IL-1β promoted HNSCC cell proliferation and migration, which indicated that macrophage-derived TNF-α and IL-1β, and not solely SPP1, were involved. VGX-1027, a compound that targets macrophages to reduce the production of TNF-α and IL-1β, was also used to verify whether SPP1 + Mac-derived TNF-α and IL-1β act on tumor cells. After treatment with VGX-1027, neither macrophages nor SPP1 + Macs promoted tumor cell growth, but when treated it solely on tumor cells, the inhibition was not significant. By extension, since both TNF-α and IL-1β are important components of NF-kappa B signaling, we considered these findings collectively with our GSVA results and presumed that the secretion of TNF-α and IL-1β by SPP1 + Macs is regulated by the NF-kappa B signaling pathway. We verified this hypothesis by treatment with PDTC, an NF-kappa B signaling inhibitor, both in vivo and in vitro. In addition to studying the effects of TNF-α and IL-1β on tumor cells, we explored their effects on adjacent macrophages. We found that TNF-α and IL-1β could increase the expression of OPN in both tumor cells and macrophages, which might be the reason for the increased number of SPP1 + Macs.

**Fig. 8:**
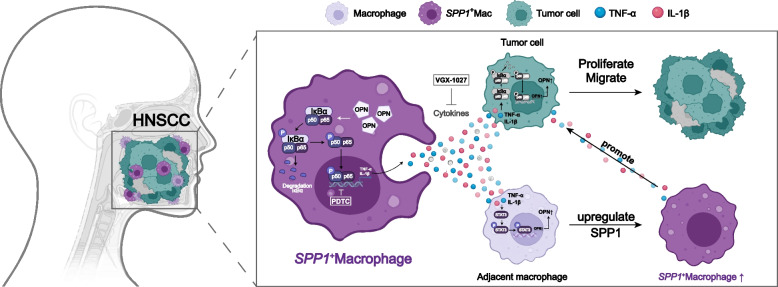
A proposed model for this study

Taken together, our findings clarify the mechanism by which SPP1 + Macs affect HNSCC cell progression (Fig. 8) and suggest that SPP1 is a promising prognostic and diagnostic marker and a potential therapeutic target for HNSCC patients. However, although the results were validated at the cellular and animal levels in this study, these experimental systems still differed from the conditions in the human TME, and thus additional validation studies in human patients are needed. In addition, in the present study, the relationships between SPP1 + Macs and other immune cells in the TIME, including CD4^+^ T cells and CD8^+^ T cells, were not elucidated; thus, our future studies will focus on these interactions.

## Conclusion

Coupled with scRNA-seq analysis and experimental validation in vivo and in vitro, our work demonstrated that SPP1^+^ Macs activate the NF-kappa B pathway to increase the secretion of TNF-α and IL-1β, which promotes the proliferation and migration of HNSCC cells. We also showed that TNF-α and IL-1β upregulate the expression of OPN in tumor cells and macrophages; thus, these factors may be candidate targets through which antitumor efficacy can be enhanced.

## Supplementary Information

Supplementary Material 1: Supplementary Figure S1. (A) Heat map showing average expression of known markers in 9 major cell types. (B) UMAP plots for 9 major cell types in adjacent tissues and tumor tissues. (C-G) UMAP plots showing the identified marker of immune cell clusters.

Supplementary Material 2: Supplementary Figure S2. (A) SPP1 expression level in normal tissues and HNSCC tissues of TCGA cohort. (B-C) SPP1 is associated with cancer stage (B) and tumor grade (C) in HNSCC of TCGA database. (D) IHC analysis of SPP1 expression in paired adjacent normal tissues and tumor tissues. Scale bars: left: 200 μm; right: 20 μm. (E) Three-line table showing the expression of SPP1 in our clinical cohort and patients’ information.

Supplementary Material 3: Supplementary Figure S3. (A) MTT assays showed SPP1-OE supernatant fasted the proliferation of HN6 and CAL27 cells. (B) Immunoblotting analysis and real-time qPCR detected SPP1 protein levels and mRNA levels in SPP1-KD macrophages (derived from THP-1 cells) respectively. (C) MTT assays showed SPP1-KD supernatant slowed the proliferation of HN6 and CAL27 cells. (D) Colony-formation abilities of HN6 and CAL27 cells were decreased when co-culture with SPP1-KD. (E–F) Migration abilities of HN6 and CAL27 cells were dropped when co-culture with SPP1-KD, which were detected using wounding-healing assays (E) and Transwell assays (F). Data are presented as the mean ± SD from three independent experiments. Scale bar, 50 μm. (G) Protein expression of IL-8 detected by ELISA between NC and OE. (H-I) The protein (H) and mRNA (I) level of TNF-α and IL-1β was elevated in OE and downregulated in KD. (*p < 0.05; **p < 0.01; ***p < 0.001; ****p < 0.0001). NC, THP-1 cells derived SPP1-NC macrophages. OE, THP-1 cells derived SPP1-OE macrophages. Sh-NC, THP-1 cells derived SPP1-Sh-NC macrophages. KD and SPP1-KD, THP-1 cells derived SPP1-KD macrophages.

Supplementary Material 4: Supplementary Figure S4. (A-C) MTT assays showed the function of different dose of rhMIF (A), rhTNF-α (B) and rhIL-1β (C) on HN6 and CAL27 cells proliferation. (D) MTT assays showed SPP1-NC supernatant with rhTNF-α or rhIL-1β facilitated the proliferation of HN6 and CAL27 cells while rhMIF slowed the proliferation. (ns, no significant difference; *p < 0.05; **p < 0.01).

Supplementary Material 5: Supplementary Figure S5. (A-H) The growth curve, colony-formation and migration ability of CAL27 cells when co-culture with SPP1-NC group (NC, NC + TNF-α, NC + IL-1β, NC + TNF-α + IL-1β), SPP1-OE group (OE, OE + VGX-1027) and SPP1-KD group (KD, KD + TNF-α, KD + IL-1β) respectively. Each group was detected using MTT assays (A, C, E), colony-formation assays (B, D, F), Transwell assays (G) and wound-healing assays (H), respectively. Scale bar, 50 μm. (*p < 0.05; **p < 0.01; ***p < 0.001; ****p < 0.0001). NC, THP-1 cells derived SPP1-NC macrophages. OE, THP-1 cells derived SPP1-OE macrophages. KD, THP-1 cells derived SPP1-KD macrophages.

Supplementary Material 6: Supplementary Figure S6. (A-P) The growth curve, colony-formation and migration ability of HN6 and CAL27 cells when coculture with SPP1-NC group (NC, NC + VGX-1027) and Control group (Ctrl, + VGX-1027) respectively. Each group was detected using MTT assays (A, C, I, K), colony-formation assays (B, D, J, L), Transwell assays (E, G, M, O) and wound-healing assays (F, H, N, P), respectively. Scale bar, 50 μm. (*p < 0.05; **p < 0.01; ***p < 0.001; ****p < 0.0001). NC, THP-1 cells derived SPP1-NC macrophages. Ctrl, tumor cells cultured with culture media without FBS.

Supplementary Material 7: Supplementary Figure S7. (A-H) The growth curve, colony-formation and migration ability of HN6 and CAL27 cells when co-culture with SPP1-OE group (OE, OE + anti-TNF-α, OE + anti-IL-1β, OE + anti-TNF-α + anti-IL-1β, OE + VGX-1027) respectively. Each group was detected using MTT assays (A, E), colony-formation assays (B, F), Transwell assays (C, G) and wound-healing assays (D, H), respectively. The concentration of anti-TNF-α and anti-IL-1β antibody was 5ug/ml. (I) Western blot was used to verify the isolation of cytoplasm and nucleus in macrophages. Scale bar, 50 μm. (*p < 0.05; **p < 0.01; ***p < 0.001; ****p < 0.0001). NC, THP-1 cells derived SPP1-NC macrophages. OE, THP-1 cells derived SPP1-OE macrophages.

Supplementary Material 8: Supplementary Figure S8. (A) The densitometry analysis of blots in HN6 and CAL27 cells, related to Fig. 6A. (B-D) The detection of PI3K/Akt (B), MAPK (C), STAT3 (D) pathway in tumor cells by western blot. (E) The densitometry analysis of blots related to Figure S8B-D. (F-I) Growth curves and colony formation and migration abilities of HN6 cells stimulated by TNF-α, IL-1β and NF-kappa B inhibitor, PDTC. MTT assays (F), colony formation assays (G), wound healing assays (H) and Transwell assays (I) were performed. Scale bar, 50 μm. (J) The MFI analysis of FCS in tumor cells, related to Fig. 6F. (K) PCR was used to measure the fold change of OPN in mRNA level in CAL27 cells. (L-N) The detection of NF-kappa B (L), PI3K/Akt (M), MAPK (N) pathway in macrophages by western blot. (O) The densitometry analysis of blots in macrophages, the upper one was related to Fig. 6J and the lower one was related to Figure S8L-N. (*p < 0.05; **p < 0.01; ***p < 0.001; ****p < 0.0001).

Supplementary Material 9.

Supplementary Material 10.

Supplementary Material 11.

Supplementary Material 12.

Supplementary Material 13.

## Data Availability

The single-cell transcriptomics data in the present study were uploaded to the Genome Sequence Archive HRA0005576 (https://ngdc.cncb.ac.cn/gsa-human/browse/HRA005576). The data generated in this study are available upon request from the corresponding author. Gene expression and clinical data are available on the TCGA HNSCC database (https://ualcan.path.uab.edu/analysis.html).

## Abbreviations

HNSCC: Head and neck squamous cell carcinoma
TIME: Tumor immune microenvironment
TAMs: Tumor-associated macrophages
SPP1^+^ Macs: SPP1^+^ macrophages
scRNA-seq: Single-cell RNA sequencing
GSVA: Gene set variation analysis
SPP1: Secreted phosphoprotein 1
OPN: Osteopontin
TNF-α: Tumor necrosis factor alpha
IL-1β: Interleukin-1 beta
