# Supplementary material for: SPP1+ macrophages promote head and neck squamous cell carcinoma progression by secreting TNF-α and IL-1β
Source: J Exp Clin Cancer Res. 2024 Dec 26;43:332. doi: 10.1186/s13046-024-03255-w (PMC11670405; doi:10.1186/s13046-024-03255-w)
Supplement: Supplementary file 1 — Supplementary Material 1: Supplementary Figure S1. (A) Heat map showing average expression of known markers in 9 major cell types. (B) UMAP plots for 9 major cell types in adjacent tissues and tumor tissues. (C-G) UMAP plots showing the identified marker of immune cell clusters. [file 13046_2024_3255_MOESM1_ESM.pdf]

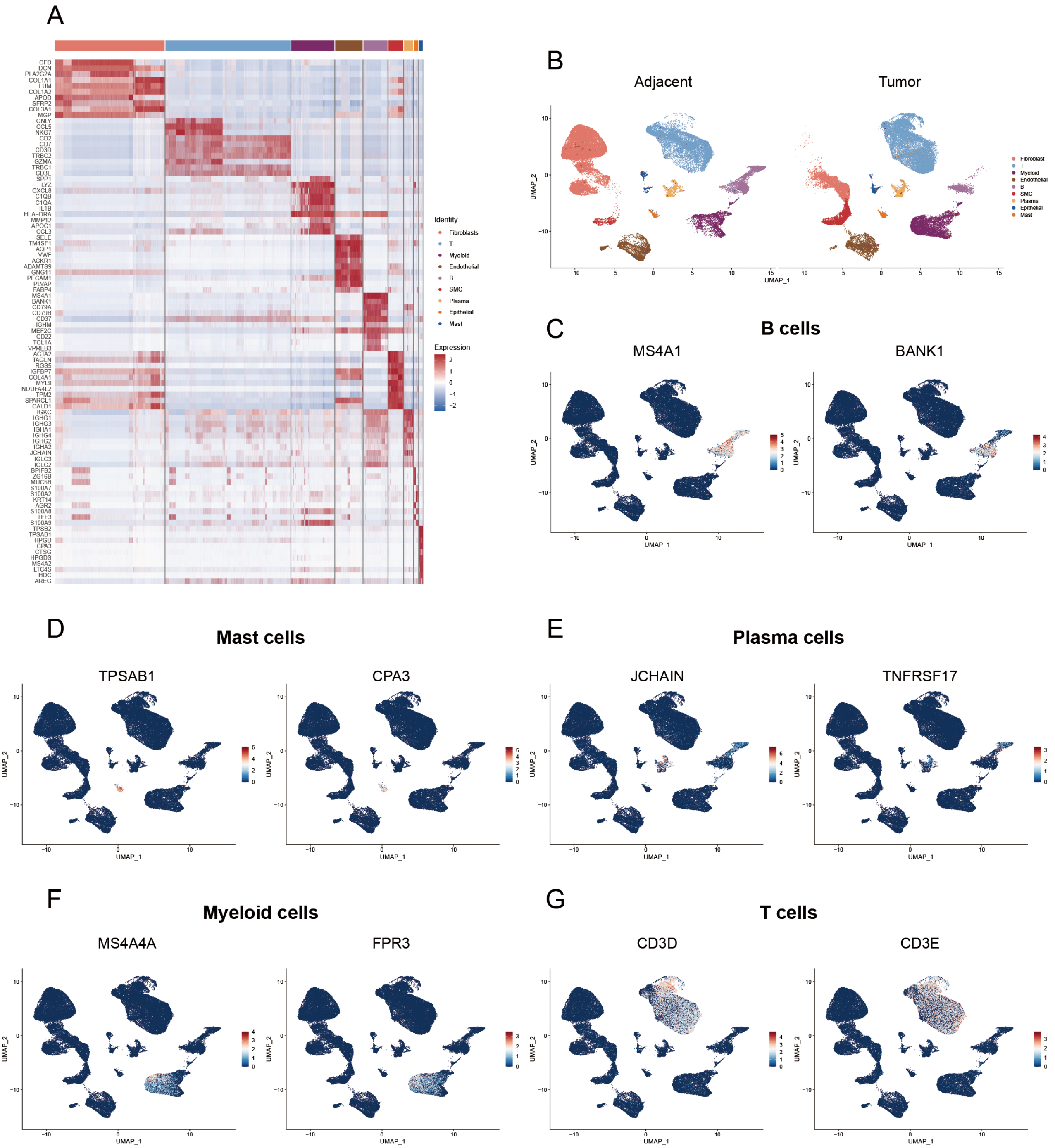
A
CFD
DCN
PLA2G2A
COL1A1
LUM
COL1A2
APOD
SFRP2
COL3A1
MGP
GNLY
CCL5
NKG7
CD2
CD7
CD3D
TRBC2
GZMA
TRBC1
CD3E
SPP1
LYZ
CXCL8
C1QB
C1QA
IL1B
HLA−DRA
MMP12
APOC1
CCL3
SELE
TM4SF1
AQP1
VWF
ACKR1
ADAMTS9
GNG11
PECAM1
PLVAP
FABP4
MS4A1
BANK1
CD79A
CD79B
CD37
IGHM
MEF2C
CD22
TCL1A
VPREB3
ACTA2
TAGLN
RGS5
IGFBP7
COL4A1
MYL9
NDUFA4L2
TPM2
SPARCL1
CALD1
IGKC
IGHG1
IGHG3
IGHA1
IGHG4
IGHG2
IGHA2
JCHAIN
IGLC3
IGLC2
BPIFB2
ZG16B
MUC5B
S100A7
S100A2
KRT14
AGR2
S100A8
TFF3
S100A9
TPSB2
TPSAB1
HPGD
CPA3
CTSG
HPGDS
MS4A2
LTC4S
HDC
AREG
Identity
Fibroblasts
T
Myeloid
Endothelial
B
SMC
Plasma
Epithelial
Mast
Expression
2
1
0
−1
−2
B
Adjacent
Tumor
Fibroblast
T
Myeloid
Endothelial
B
SMC
Plasma
Epithelial
Mast
UMAP_1
UMAP_2
C
B cells
MS4A1
BANK1
D
Mast cells
TPSAB1
CPA3
E
Plasma cells
JCHAIN
TNFRSF17
F
Myeloid cells
MS4A4A
FPR3
G
T cells
CD3D
CD3E
