# Supplementary material for: SPP1+ macrophages promote head and neck squamous cell carcinoma progression by secreting TNF-α and IL-1β
Source: J Exp Clin Cancer Res. 2024 Dec 26;43:332. doi: 10.1186/s13046-024-03255-w (PMC11670405; doi:10.1186/s13046-024-03255-w)

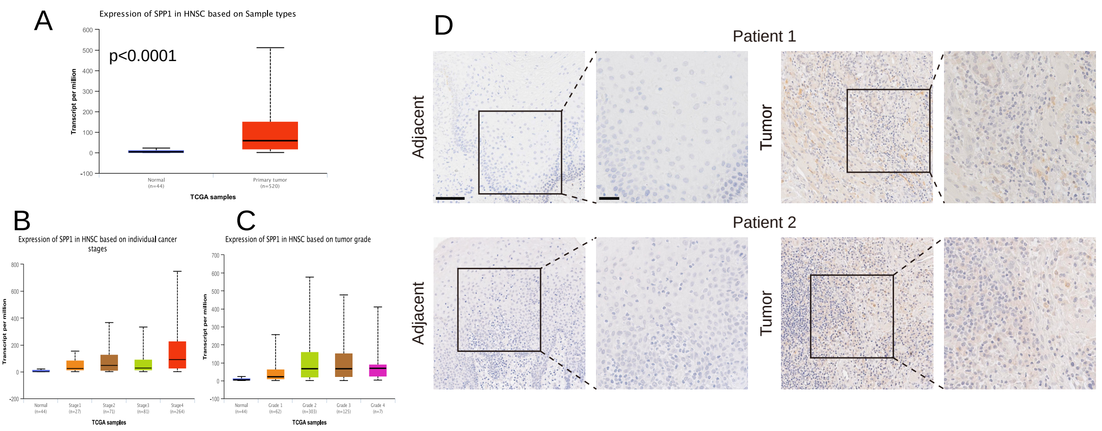

E

Characteristics	No. of Patients No.	%	OPN expression ($2^{-\Delta Ct}$ Mean±SD)	P value
**Age (years)**				0.8547
≥60	49	66.22	3.477±5.489	
<60	25	33.78	3.230±5.438	
**Gender**				0.418
Male	47	63.51	3.785±5.761	
Female	27	36.49	2.713±4.846	
**Smoking history**				0.2823
Nonsmoker	55	74.32	3.796±5.908	
Smoker	19	25.68	2.230±3.632	
**Alcohol history**				0.9084
Nondrinker	57	77.03	3.354±5.492	
Drinker	17	22.97	3.528±5.404	
**Tumor size (cm)**				**0.0403**
≤4	12	16.22	0.4615±0.8151	
>4	62	83.78	3.961±5.764	
**Lymph node metastasis**				0.5513
pN1 to pN2	40	54.05	3.043±4.757	
pN0	34	45.95	3.806±6.188	
**TNM stage**				0.2296
Ⅰ	8	10.81	0.087±0.117	
Ⅱ	23	31.08	4.650±6.397	
Ⅲ	16	21.62	2.937±4.565	
Ⅳ	27	36.49	3.574±5.601	
**Pathological differentiation**				0.5713
Well	10	13.51	4.307±6.871	
Moderately/poorly	64	86.49	3.251±5.228	
**Disease Site**				0.3153
Tongue	21	28.38	4.322±6.231	
Gingiva	14	18.92	5.386±7.340	
Cheek	19	25.68	1.660±2.707	
Floor of Mouth	8	10.81	2.685±4.157	
Oropharynx	12	16.22	2.664±5.097	
**Tumor type**				0.3525
Primary	61	82.43	3.667±5.652	
Recurrence	13	17.57	2.112±4.224	
**Prior Radiotherapy**				0.7072
Yes	15	20.27	2.918±4.631	
No	59	79.73	3.515±5.651	

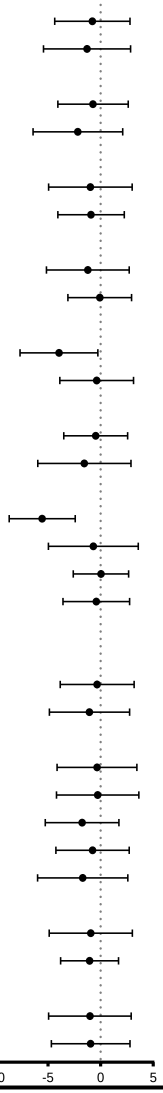

Supplement: Supplementary file 2 — Supplementary Material 2: Supplementary Figure S2. (A) SPP1 expression level in normal tissues and HNSCC tissues of TCGA cohort. (B-C) SPP1 is associated with cancer stage (B) and tumor grade (C) in HNSCC of TCGA database. (D) IHC analysis of SPP1 expression in paired adjacent normal tissues and tumor tissues. Scale bars: left: 200 μm; right: 20 μm. (E) Three-line table showing the expression of SPP1 in our clinical cohort and patients’ information. [file 13046_2024_3255_MOESM2_ESM.pdf]
