# Supplementary figures and images for: SPP1+ macrophages promote head and neck squamous cell carcinoma progression by secreting TNF-α and IL-1β
Source: J Exp Clin Cancer Res. 2024 Dec 26;43:332. doi: 10.1186/s13046-024-03255-w (PMC11670405; doi:10.1186/s13046-024-03255-w)

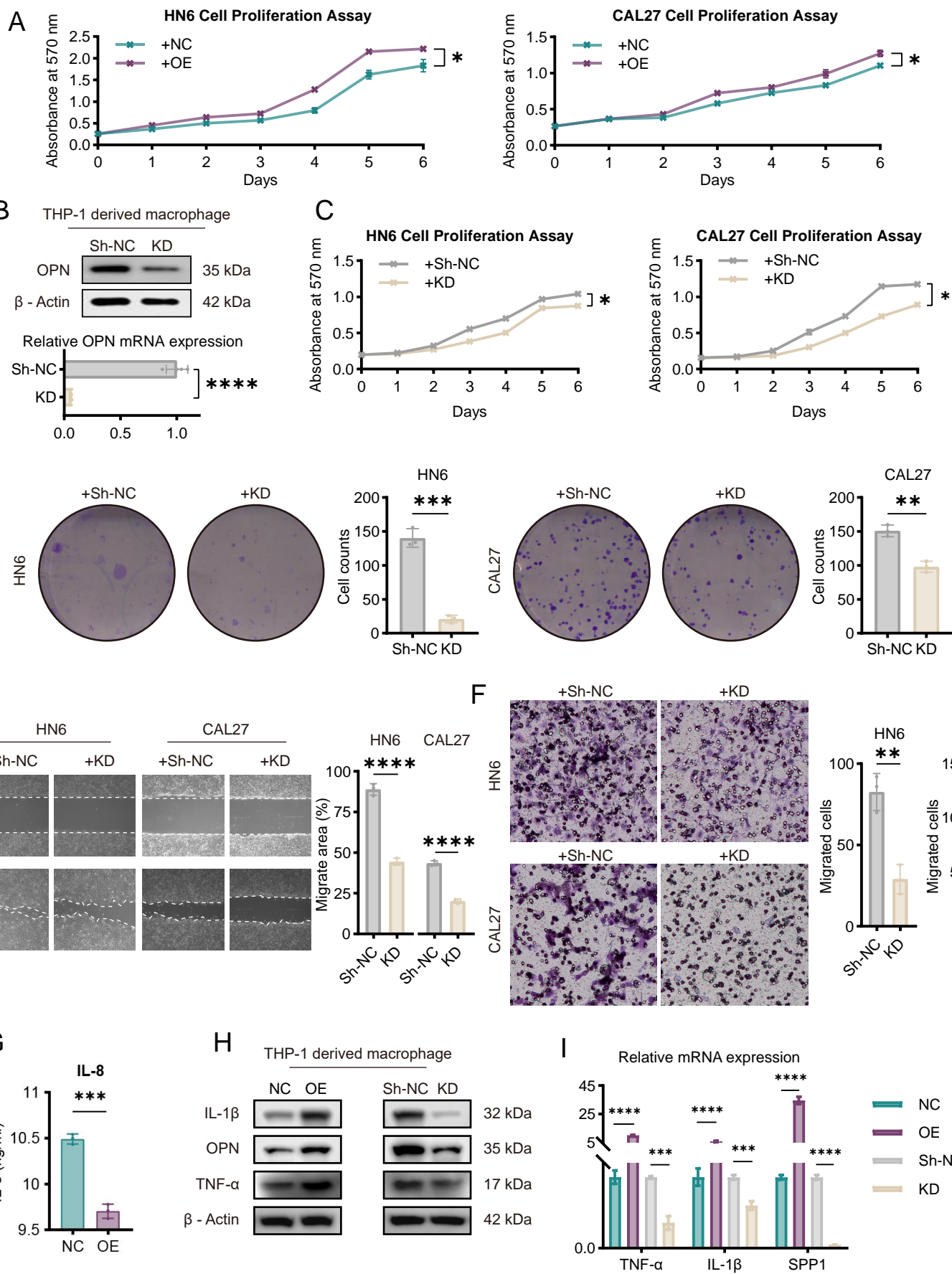

Supplement: Supplementary file 3 — Supplementary Material 3: Supplementary Figure S3. (A) MTT assays showed SPP1-OE supernatant fasted the proliferation of HN6 and CAL27 cells. (B) Immunoblotting analysis and real-time qPCR detected SPP1 protein levels and mRNA levels in SPP1-KD macrophages (derived from THP-1 cells) respectively. (C) MTT assays showed SPP1-KD supernatant slowed the proliferation of HN6 and CAL27 cells. (D) Colony-formation abilities of HN6 and CAL27 cells were decreased when co-culture with SPP1-KD. (E–F) Migration abilities of HN6 and CAL27 cells were dropped when co-culture with SPP1-KD, which were detected using wounding-healing assays (E) and Transwell assays (F). Data are presented as the mean ± SD from three independent experiments. Scale bar, 50 μm. (G) Protein expression of IL-8 detected by ELISA between NC and OE. (H-I) The protein (H) and mRNA (I) level of TNF-α and IL-1β was elevated in OE and downregulated in KD. (*p < 0.05; **p < 0.01; ***p < 0.001; ****p < 0.0001). NC, THP-1 cells derived SPP1-NC macrophages. OE, THP-1 cells derived SPP1-OE macrophages. Sh-NC, THP-1 cells derived SPP1-Sh-NC macrophages. KD and SPP1-KD, THP-1 cells derived SPP1-KD macrophages. [file 13046_2024_3255_MOESM3_ESM.pdf]

A

MIF

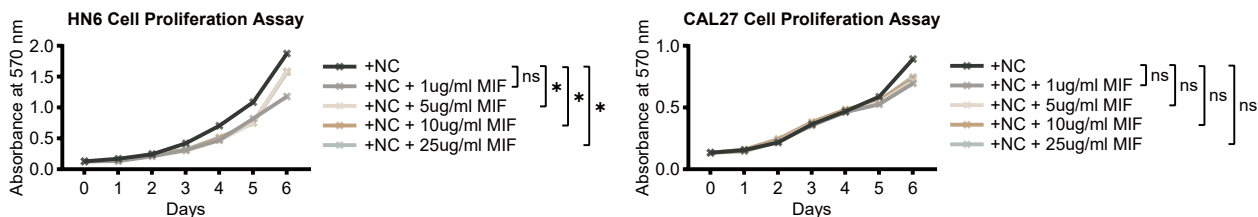

B

TNF- $\alpha$ 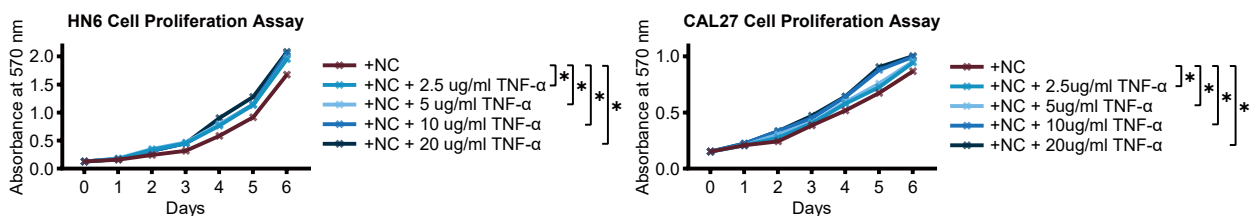

C

IL-1 $\beta$ 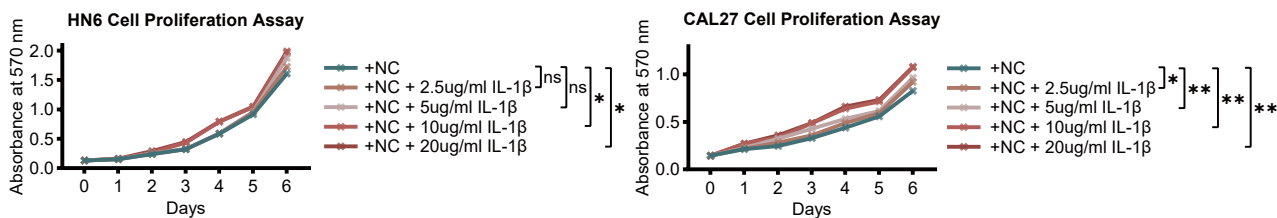

D

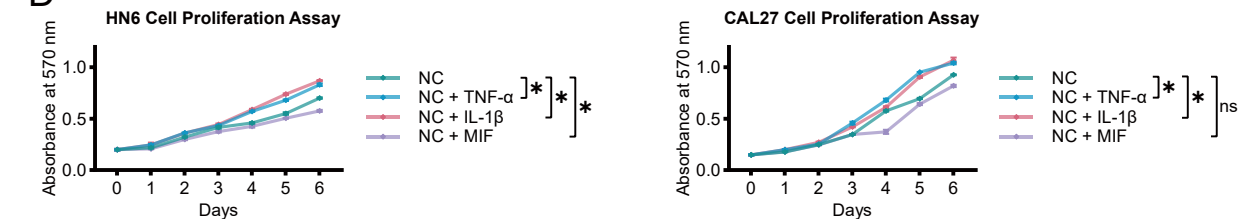

Supplement: Supplementary file 4 — Supplementary Material 4: Supplementary Figure S4. (A-C) MTT assays showed the function of different dose of rhMIF (A), rhTNF-α (B) and rhIL-1β (C) on HN6 and CAL27 cells proliferation. (D) MTT assays showed SPP1-NC supernatant with rhTNF-α or rhIL-1β facilitated the proliferation of HN6 and CAL27 cells while rhMIF slowed the proliferation. (ns, no significant difference; *p < 0.05; **p < 0.01). [file 13046_2024_3255_MOESM4_ESM.pdf]

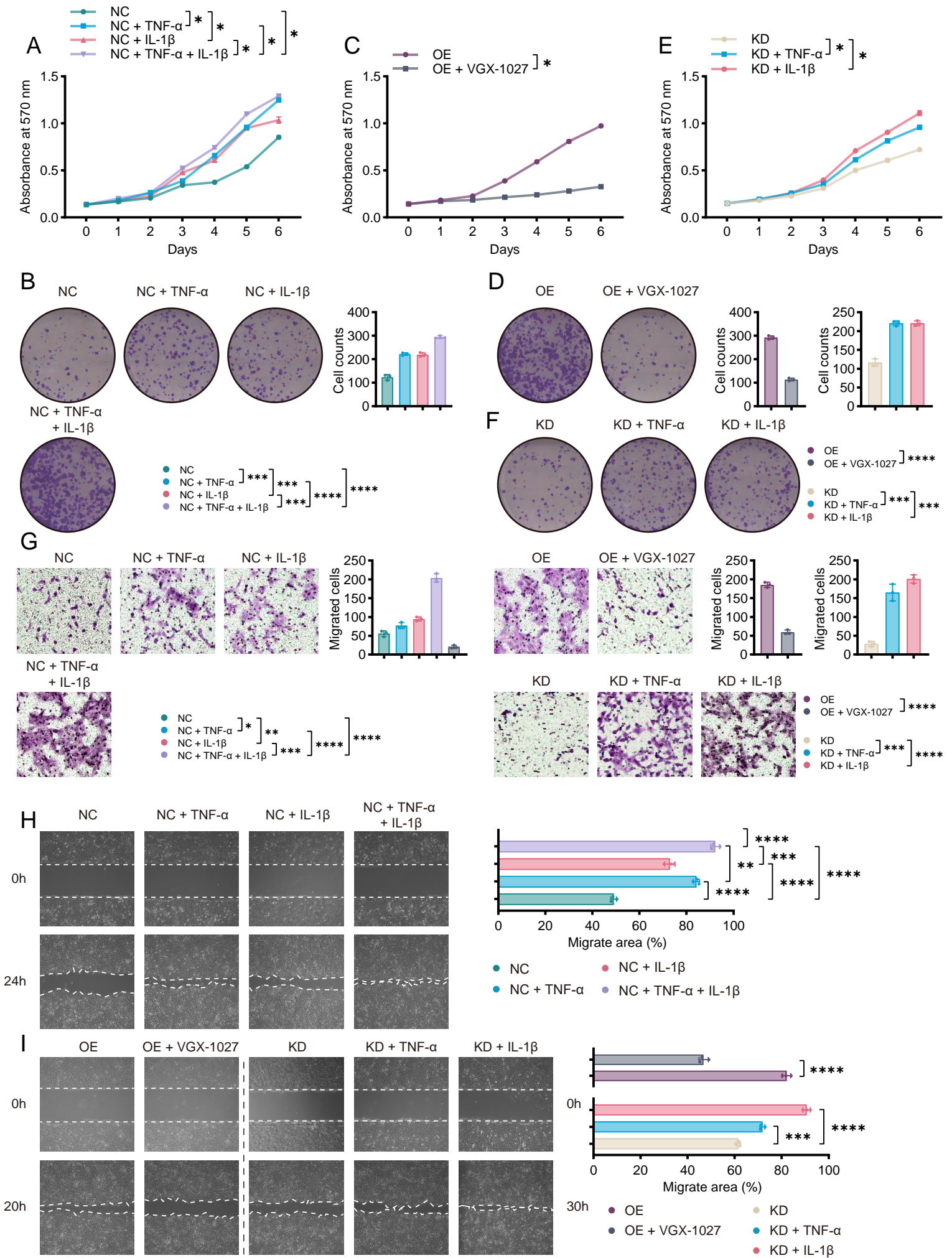

Supplement: Supplementary file 5 — Supplementary Material 5: Supplementary Figure S5. (A-H) The growth curve, colony-formation and migration ability of CAL27 cells when co-culture with SPP1-NC group (NC, NC + TNF-α, NC + IL-1β, NC + TNF-α + IL-1β), SPP1-OE group (OE, OE + VGX-1027) and SPP1-KD group (KD, KD + TNF-α, KD + IL-1β) respectively. Each group was detected using MTT assays (A, C, E), colony-formation assays (B, D, F), Transwell assays (G) and wound-healing assays (H), respectively. Scale bar, 50 μm. (*p < 0.05; **p < 0.01; ***p < 0.001; ****p < 0.0001). NC, THP-1 cells derived SPP1-NC macrophages. OE, THP-1 cells derived SPP1-OE macrophages. KD, THP-1 cells derived SPP1-KD macrophages. [file 13046_2024_3255_MOESM5_ESM.pdf]

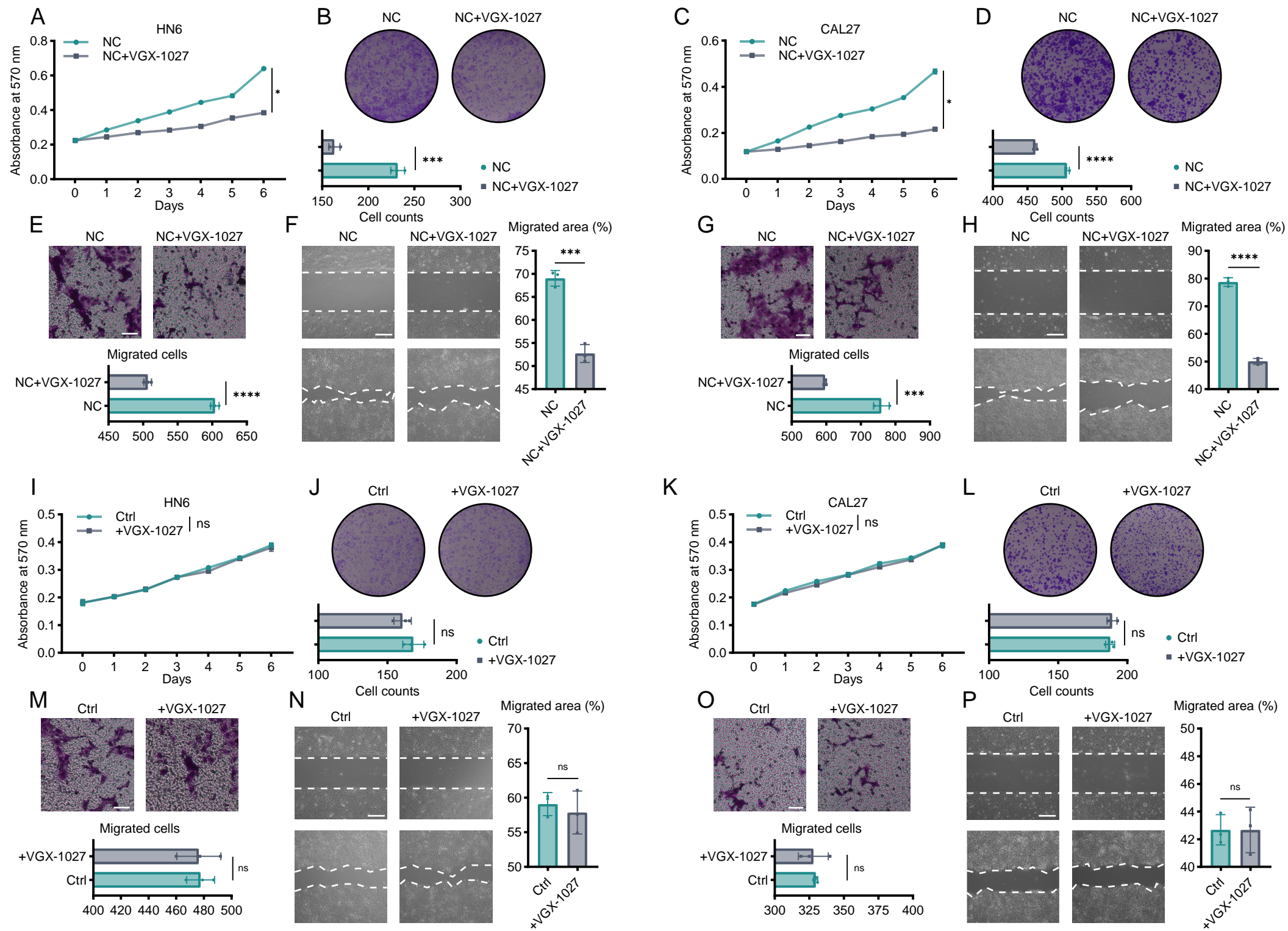

Supplement: Supplementary file 6 — Supplementary Material 6: Supplementary Figure S6. (A-P) The growth curve, colony-formation and migration ability of HN6 and CAL27 cells when coculture with SPP1-NC group (NC, NC + VGX-1027) and Control group (Ctrl, + VGX-1027) respectively. Each group was detected using MTT assays (A, C, I, K), colony-formation assays (B, D, J, L), Transwell assays (E, G, M, O) and wound-healing assays (F, H, N, P), respectively. Scale bar, 50 μm. (*p < 0.05; **p < 0.01; ***p < 0.001; ****p < 0.0001). NC, THP-1 cells derived SPP1-NC macrophages. Ctrl, tumor cells cultured with culture media without FBS. [file 13046_2024_3255_MOESM6_ESM.pdf]

## HN6

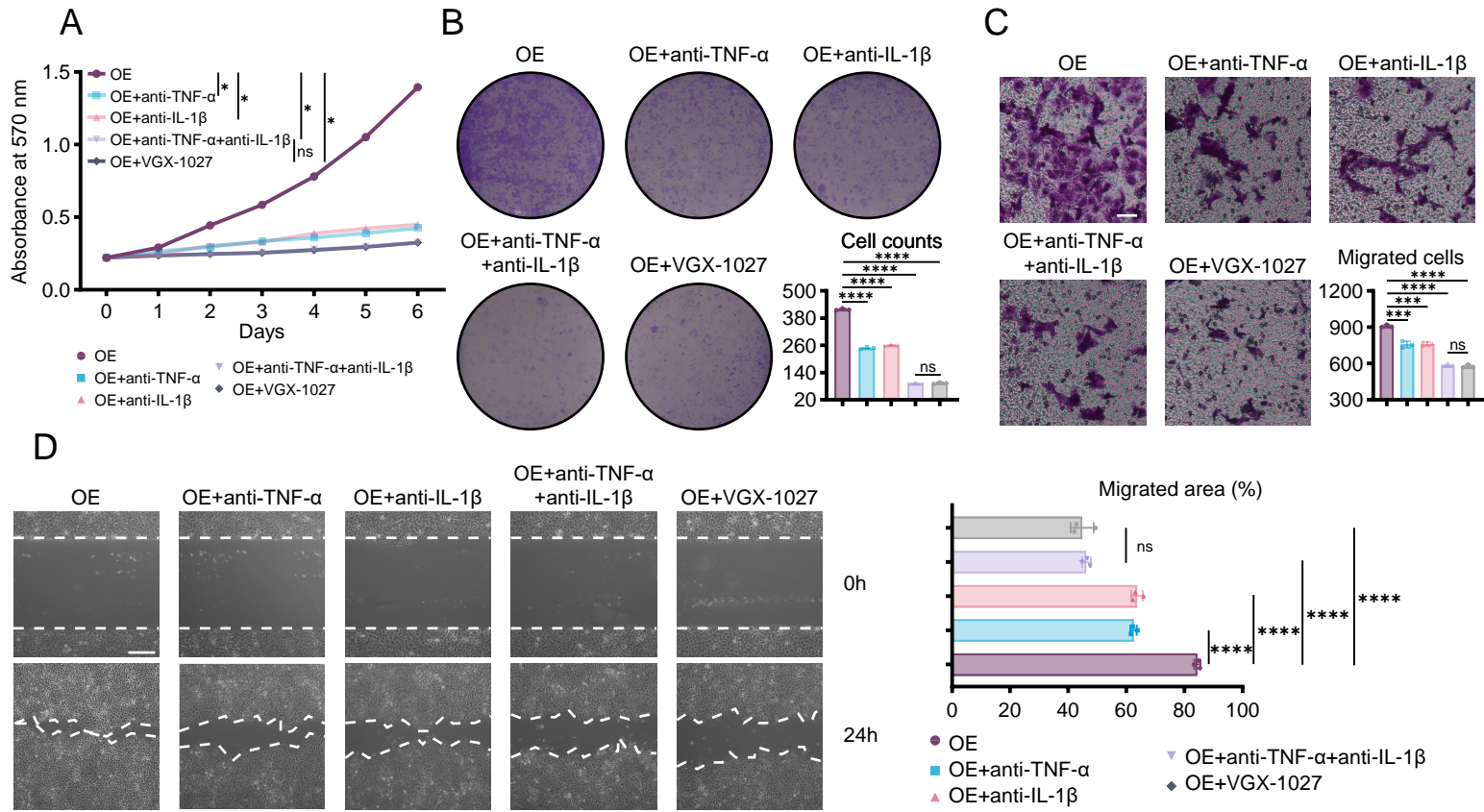

## CAL27

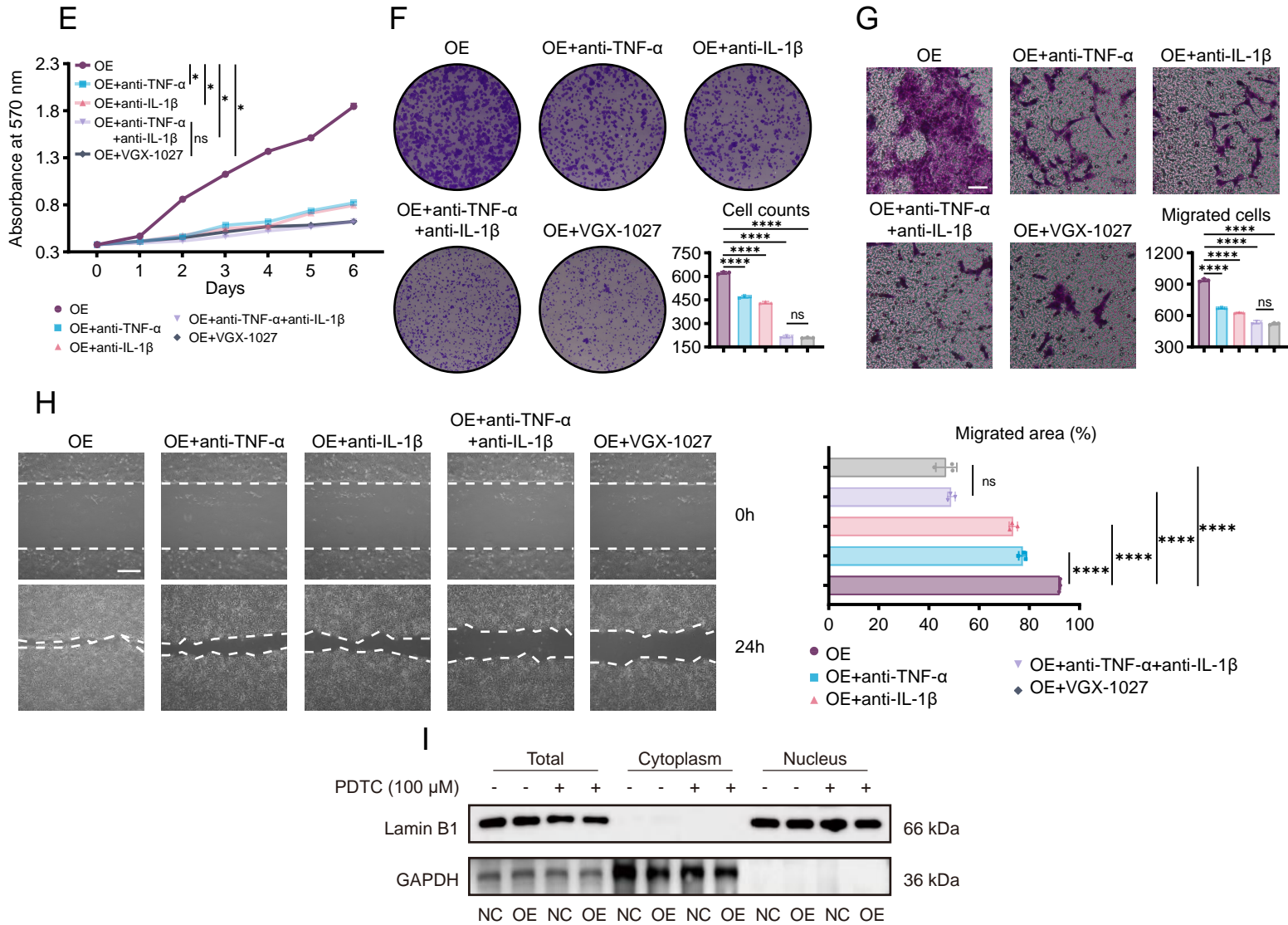

Supplement: Supplementary file 7 — Supplementary Material 7: Supplementary Figure S7. (A-H) The growth curve, colony-formation and migration ability of HN6 and CAL27 cells when co-culture with SPP1-OE group (OE, OE + anti-TNF-α, OE + anti-IL-1β, OE + anti-TNF-α + anti-IL-1β, OE + VGX-1027) respectively. Each group was detected using MTT assays (A, E), colony-formation assays (B, F), Transwell assays (C, G) and wound-healing assays (D, H), respectively. The concentration of anti-TNF-α and anti-IL-1β antibody was 5ug/ml. (I) Western blot was used to verify the isolation of cytoplasm and nucleus in macrophages. Scale bar, 50 μm. (*p < 0.05; **p < 0.01; ***p < 0.001; ****p < 0.0001). NC, THP-1 cells derived SPP1-NC macrophages. OE, THP-1 cells derived SPP1-OE macrophages. [file 13046_2024_3255_MOESM7_ESM.pdf]

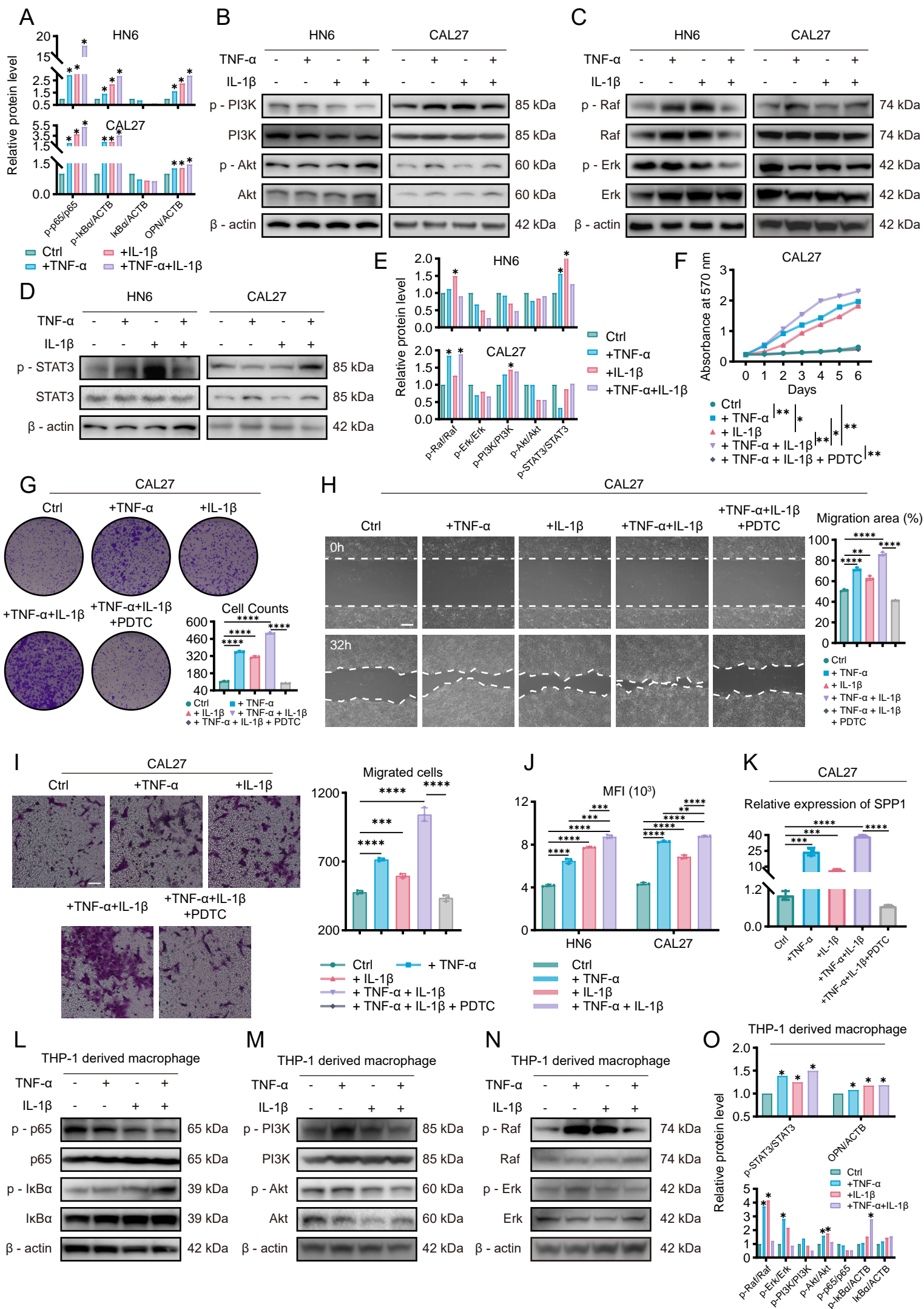

Supplement: Supplementary file 8 — Supplementary Material 8: Supplementary Figure S8. (A) The densitometry analysis of blots in HN6 and CAL27 cells, related to Fig. 6A. (B-D) The detection of PI3K/Akt (B), MAPK (C), STAT3 (D) pathway in tumor cells by western blot. (E) The densitometry analysis of blots related to Figure S8B-D. (F-I) Growth curves and colony formation and migration abilities of HN6 cells stimulated by TNF-α, IL-1β and NF-kappa B inhibitor, PDTC. MTT assays (F), colony formation assays (G), wound healing assays (H) and Transwell assays (I) were performed. Scale bar, 50 μm. (J) The MFI analysis of FCS in tumor cells, related to Fig. 6F. (K) PCR was used to measure the fold change of OPN in mRNA level in CAL27 cells. (L-N) The detection of NF-kappa B (L), PI3K/Akt (M), MAPK (N) pathway in macrophages by western blot. (O) The densitometry analysis of blots in macrophages, the upper one was related to Fig. 6J and the lower one was related to Figure S8L-N. (*p < 0.05; **p < 0.01; ***p < 0.001; ****p < 0.0001). [file 13046_2024_3255_MOESM8_ESM.pdf]
